# A supervised learning method for classifying methylation disorders

**DOI:** 10.1186/s12859-024-05673-1

**Published:** 2024-02-12

**Authors:** Jesse R. Walsh, Guangchao Sun, Jagadheshwar Balan, Jayson Hardcastle, Jason Vollenweider, Calvin Jerde, Kandelaria Rumilla, Christy Koellner, Alaa Koleilat, Linda Hasadsri, Benjamin Kipp, Garrett Jenkinson, Eric Klee

**Affiliations:** 1 Mayo Clinic, Rochester, MN USA; 2 Department of Molecular and Medical Genetics, Oregon Health and Science University, Portland, OR USA

**Keywords:** Congenital disease, Methylation, Diagnosis, Machine learning, Angelman syndrome, Prader–Willi syndrome, Beckwith–Wiedemann syndrome, Silver–Russell syndrome, Russell–Silver syndrome

## Abstract

**Background:**

DNA methylation is one of the most stable and well-characterized epigenetic alterations in humans. Accordingly, it has already found clinical utility as a molecular biomarker in a variety of disease contexts. Existing methods for clinical diagnosis of methylation-related disorders focus on outlier detection in a small number of CpG sites using standardized cutoffs which differentiate healthy from abnormal methylation levels. The standardized cutoff values used in these methods do not take into account methylation patterns which are known to differ between the sexes and with age.

**Results:**

Here we profile genome-wide DNA methylation from blood samples drawn from within a cohort composed of healthy controls of different age and sex alongside patients with Prader–Willi syndrome (PWS), Beckwith–Wiedemann syndrome, Fragile-X syndrome, Angelman syndrome, and Silver–Russell syndrome. We propose a Generalized Additive Model to perform age and sex adjusted outlier analysis of around 700,000 CpG sites throughout the human genome. Utilizing z-scores among the cohort for each site, we deployed an ensemble based machine learning pipeline and achieved a combined prediction accuracy of 0.96 (Binomial 95% Confidence Interval 0.868$-$0.995).

**Conclusion:**

We demonstrate a method for age and sex adjusted outlier detection of differentially methylated loci based on a large cohort of healthy individuals. We present a custom machine learning pipeline utilizing this outlier analysis to classify samples for potential methylation associated congenital disorders. These methods are able to achieve high accuracy when used with machine learning methods to classify abnormal methylation patterns.

## Background

DNA methylation is a form of epigenetic modification that occurs in humans primarily through the addition of a methyl group to the cytosine of CpG dinucleotide sequences [1]. Methylation patterns in mammals are heritable, as they are passed from parent to offspring through the process of imprinting [2]. The human genome is depleted for CpG dinucleotides with an estimated 28.3 million CpG sites in the human genome [3], most of which are thought to be methylated in somatic tissues [4, 5]. Clusters of conserved CpG sites, known as CpG islands, are found in most gene promoter regions [6]. These have been associated with gene regulation by acting as repressors of gene expression when methylated [7]. Methylation patterns are known to differ between the sexes and over the lifetime of an individual. Large differences in methylation of the sex chromosomes in males and females are observed due to X chromosome inactivation in females during early embryonic development [4], while smaller differences in expression between the sexes have been observed on the autosomes in certain tissues [8]. Methylation patterns in a set of 353 specific CpG sites have been proposed as a predictor of chronological age [9], and changes in methylation are intricately involved in tissue differentiation and human development [10, 11].

Abnormal methylation can be associated with disorders in humans. Some imprinting disorders are relatively well characterized to have local methylation abnormalities at a small number of known CpG sites such as Prader–Willi syndrome (PWS), Beckwith–Wiedemann syndrome (BWS), Fragile-X syndrome (FXS), and Angelman syndrome (AS) [12]. Other disorders, such as Silver–Russell syndrome (SRS), are associated with broad and non-specific disruptions to methylation patterns at a specific chromosome or throughout the genome [13]. These are generally syndromic, associated with developmental abnormalities, and diagnosed in young children. The clinical gold standard in tests for methylation abnormalities is Methylation-Specific Multiplex Ligation-Dependent Probe Amplification (MS-MLPA) [14, 15]. While it has demonstrated clinical utility, the throughput of this approach is limited by the number of probes in the array and incomplete knowledge of probe associations with certain disorders. In addition, results can be confounded by DNA contamination, benign point mutations, and gene copy number variations [15]. Array-based methods offer an alternative approach to methylation detection, and include the Illumina Infinium HumanMethylation450 BeadChip and the EPIC BeadChip (850k) arrays. The EPIC array measures over 850,000 of the estimated 28 million CpG sites in the human genome while covering a wide range of genomic categories including CpG islands, shores, shelves, genes, regulatory elements, and more [16–18]. This allows for profiling both genome-wide methylation patterns as well as the methylation level of specific genomic regions known to be associated with certain disorders.

Methylation levels at certain genomic loci in the human genome are highly dynamic depending on age, sex, and tissue type [9, 10]. This can interfere with outlier detection for probes that vary between the sexes or with age if those factors are not adjusted for. A conventional approach to outlier detection for a given probe would involve a group vs. group comparison between a control group and a cohort of samples with a confirmed disorder. In a clinical setting, this approach often leads to developing reference methylation ranges for a small set of probes which are then used to detect abnormal methylation levels either above or below the reference. A common limitation of this approach is the reliance on a single reference range based on the entire cohort without incorporating potential probe level effects of biological age or sex. Here we performed a genome-wide methylation study using the Illumina EPIC BeadChip (850K) within a cohort including 149 healthy controls with a wide age range and an even male-to-female ratio. We describe a Generalized Additive Model (GAM) which uses the control cohort to build probe level age and sex stratified methylation models which can robustly reduce false negatives for outlier identification in 134 patients clinically diagnosed for imprinting disorders. Lastly, using z-scores from a combination of probes with known association with epigenetic disorders and extracted global features, we trained an ensemble based classifier that achieved a prediction accuracy of 0.96.

## Results

### Patient cohort and sample processing

**Fig. 1:**
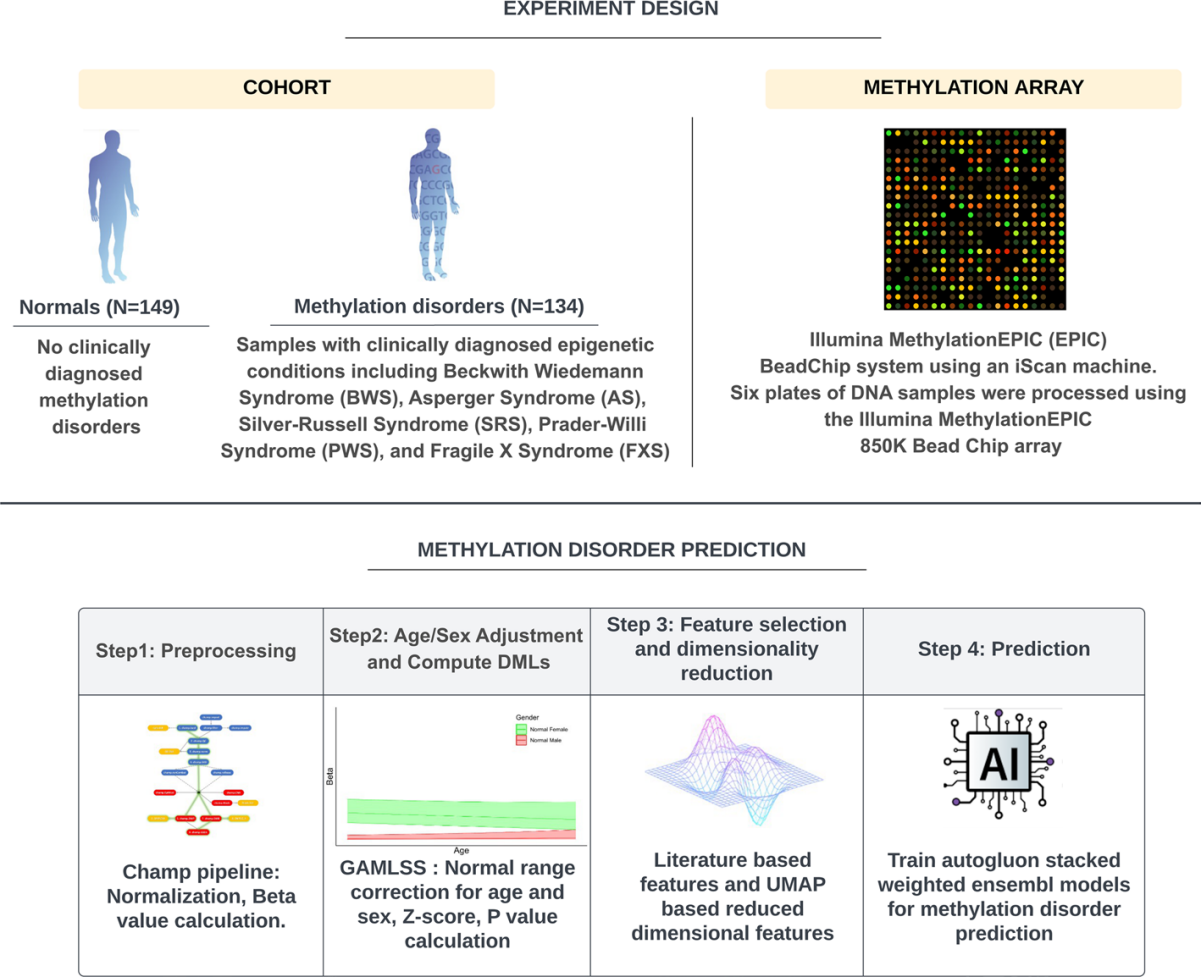
Experimental design and analysis workflow. Cohort includes 149 controls and 134 abnormal samples with a variety of epigenetic disorders. These samples were analyzed using the Illumina MethylationEPIC (850k) system on an iScan machine. After preprocessing using the Champ pipeline (image reproduced in part from [20]), an age and sex adjusted reference model was created from the normal samples to compute z-scores of probes in all samples. Z-scores of disease associated probes and UMAP coefficients computed from the z-score matrix of the remaining probes were used as global features in the machine learning algorithm AutoGluon to classify epigenetic disorders

**Fig. 2:**
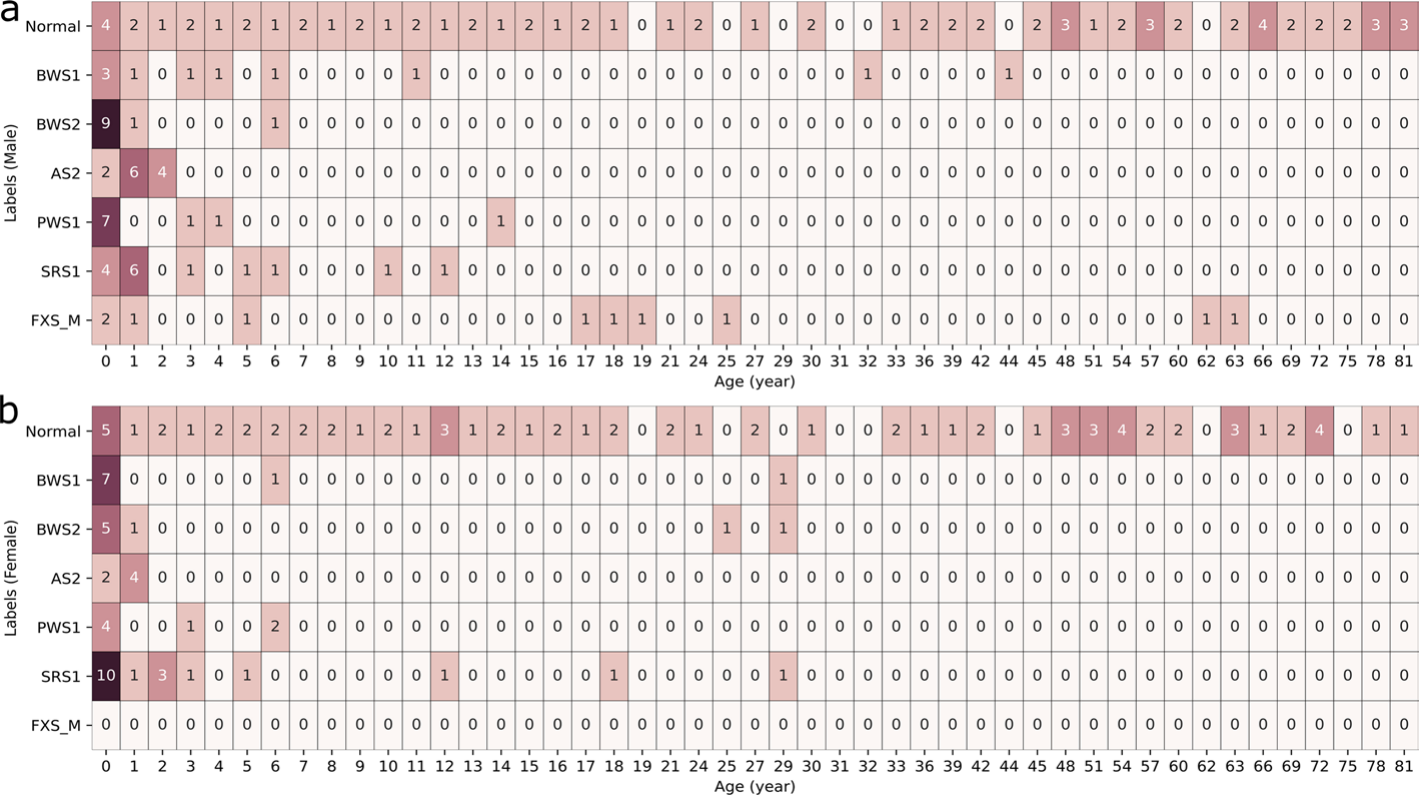
Heterogeneity of the cohort based on age and sex. (**a**) Number of samples for normal and congenital disease categories in male samples. (**b**) Number of samples for normal and congenital disease categories in female samples

A patient cohort of 283 research consented patients were selected for this study from existing patients at Mayo Clinic. There were two subgroups in this cohort. The first subgroup included 134 abnormal samples with clinically diagnosed (via MS-MLPA) epigenetic disorders including Beckwith–Wiedemann syndrome, Angelman syndrome, Silver–Russell syndrome, Prader–Willi syndrome, and Fragile X syndrome (Fig. 1). The BWS group was further divided into BWS1 (exhibiting H19 Hypermethylation and LIT1 Hypomethylation) and BWS2 (exhibiting H19 Normal methylation and LIT1 Hypomethylation) (See Table 1). The second subgroup consisted of 149 controls that had no diagnosed or suspected epigenetic disorders based on results in their medical records. All control samples were screened by MS-MLPA assay to confirm a negative result for any of the abnormal methylation conditions in this study. Control samples included both males and females with age ranges from 1 day old to 81 years old (Fig. 2). Samples were derived from whole blood and were processed using on the Illumina Infinium MethylationEPIC BeadChip 850K Array [18] across six plates. The package ChAMP (v2.20.1) [19, 20] was used in R (v4.0.3) to perform quality control, BMIQ normalization, and whole blood admixture adjustment using default parameters. This resulted in beta values representing qualitative methylation levels for about 746,834 CpG sites. CpG site annotations were retrieved using the R package IlluminaHumanMethylationEPICanno.ilm10b4.hg19 (v0.6.0).

### Whole genome methylation profiling confirms expected hyper- and hypomethylation in disease associated loci

**Fig. 3:**
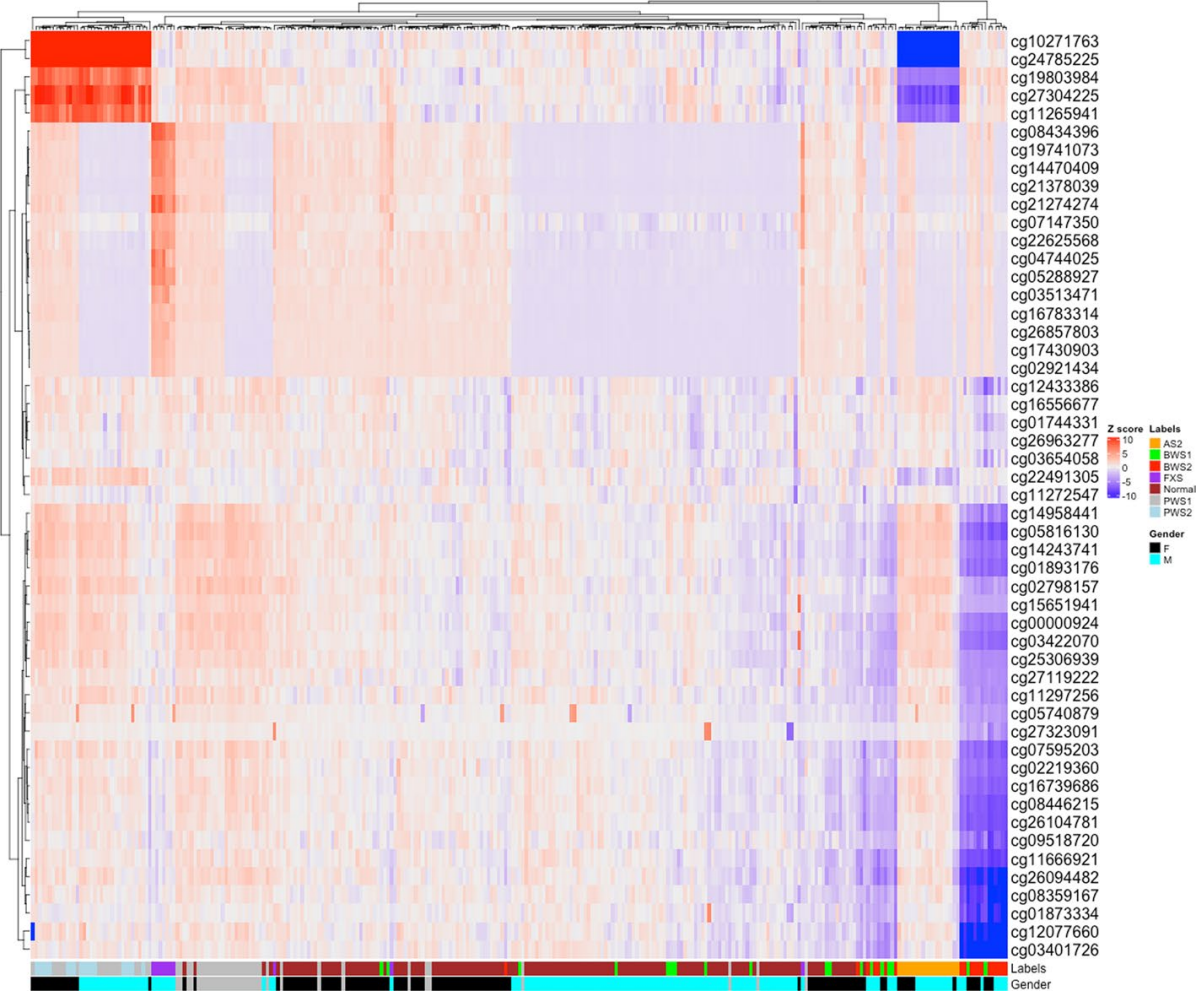
Clustering based on unadjusted z-scores. Probes known to be involved in the abnormalities included in this study are shown. The z-scores were calculated using the normalized probe beta values of the samples and were plotted and clustered using k-means method

Variation in methylation patterns for several genomic regions has been associated with specific imprinting disorders. In order to confirm expected patterns of hypo- and hyper-methylation exist in our data in clinically relevant genomic regions, we first extracted the list of probes overlapped by the regions associated with BWS, PWS, AS, and FXS (Additional file 1: Table S1). We then examined the unadjusted methylation beta values by performing unsupervised clustering using the k-nearest neighbors method. We confirmed several expected patterns (Fig. 3), including a cluster of several probes in the SNRPN/SNURF locus which show hypermethylation in PWS samples and hypomethylation in the AS samples relative to the normal samples [21, 22], a cluster including several probes related to the FMR1 promoter locus which are hypermethylated in FXS male samples relative to the normal samples [23], and a cluster of several probes from the KCNQ10T1 locus which showed hypomethylation in the BWS samples relative to the normal samples [15, 24, 25].

Observing these patterns in our data confirms the utility of these loci for detecting the presence of at least some of the imprinting disorders of interest in this study. However, there is no confirmed effective probes to detect SRS and it has been reported that the heterogeneity is high among SRS patients [26]. To investigate whether SRS patients show unified variation of methylation at the global level, we extracted 30 UMAP dimensions from the highly variable probes of which the methylation level showing standard deviation higher than 1.5 and plotted the UMAP dimensions that separate SRS patients from other samples (Mann–Whitney U test *p* $\le$ 0.00001), however, no obvious separation was observed (Additional file 1: Fig. S1). K-means clustering performed directly with the highly variable probes did not cluster SRS patients into one either, further proving the high heterogeneity of SRS patients (Additional file 1: Fig. S2).

In order to assess the benefit of incorporating information from the whole-genome methylation levels for detecting imprinting disorders, we performed a principal component analysis of the methylation data. The first 10 principal components extracted from 109,131 probes exhibiting substantial deviations from the normal range together explained 56 percent of the total variance in the dataset (Additional file 1: Table S2). A scatter plot of principal components 1 and 2 (PC1 and PC2) explained 27.5 and 12.7 percent respectively of the total variance, however these principal components alone did not show obvious separation of age or sex. Including PC8, PC9 and PC10 clearly captured global variation due to sex (Additional file 1: Fig. S3), while PC7 seemed to capture global methylation variation due to age range (Additional file 1: Fig. S4). These results suggested that sex and age both contributed to global variations of methylation level in the cohorts used in this study. Age and sex effects may or may not be statistically significant depending on the specific probe. Several examples showing beta values by sex over age are provided in (Additional file 1: Fig. S5).

### Age and sex adjustment increase reliability of clinically relevant probes

Methylation disorders associated with genomic loci are influenced by the combined effect of the methylation levels of all the CpG sites in the region. If enough of the CpG sites are hyper- or hypomethylated in unison, dysregulation of the genes may occur, and the patient may present with symptoms of the disorder. This implies that the individual methylation level of a single CpG site may or may not agree with the other CpG sites in the locus. We assessed the sensitivity of the probes in the BWS locus by comparing outlier status of individual probes before and after adjustment. Here we define probe sensitivity as the number of confirmed BWS samples in which the z-score of a relevant probe is detected as an outlier (z-score $\ge$ 3 or $\le$ −3).

**Fig. 4:**
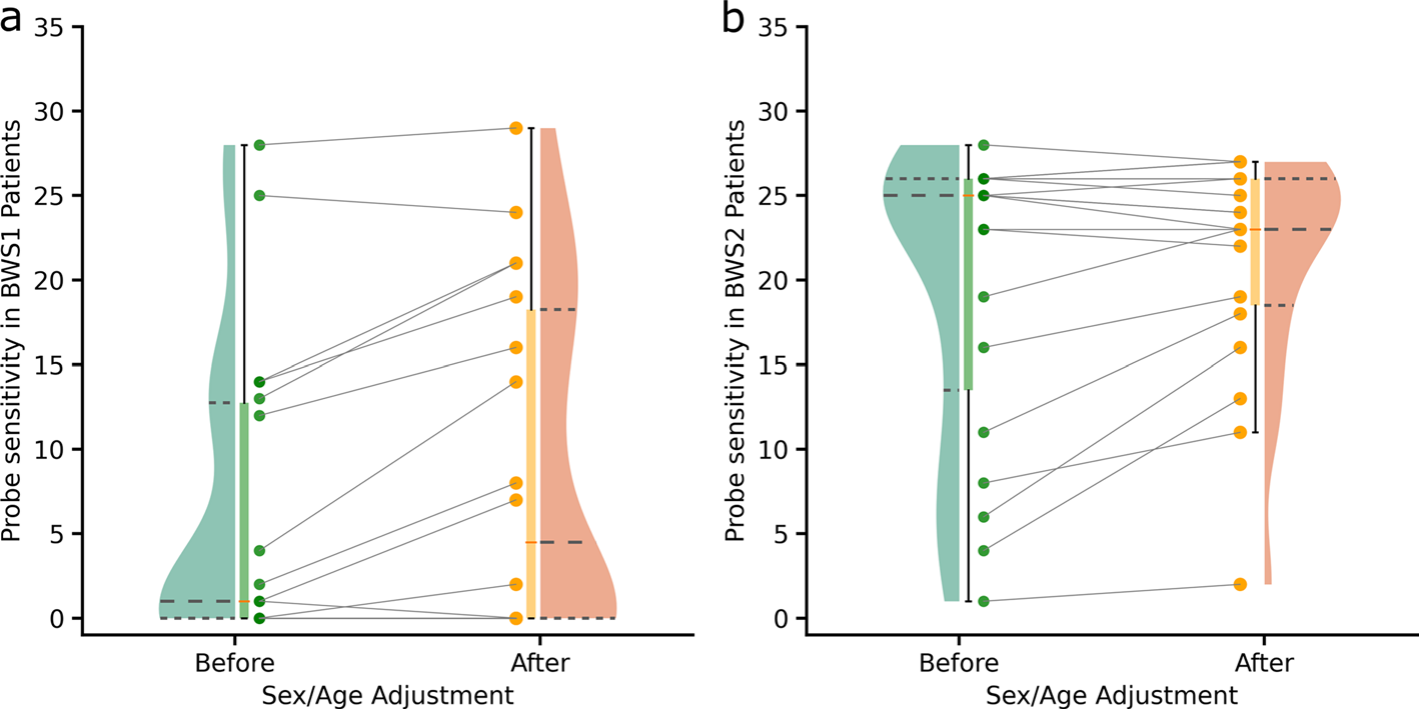
Adjusting normal ranges for age and sex increases reliability of outlier identification. (**a)** The counts of BWS1 associated probes being identified as outliers in the samples labeled as BWS1 before and after age and sex adjustment of normal methylation range.  (**b**) The counts of BWS2 associated probes being identified as outliers in the samples labeled as BWS2 before and after age and sex adjustment of normal methylation range

There are 31 probes overlapping the KCNQ10T1 region. For both BWS1 (Fig. 4a) and BWS2 (Fig. 4b) samples, we identified samples in which fewer than 5 out of 31 probes shown as outliers, which could lead to false negative diagnosis. With the adjusted z-scores, the number of outlier probes in these samples were increased, which reduced the chance for false negative diagnosis. When we cluster the disease-associated probes based on the adjusted z-scores using nearest neighbor method, the expected hyper- or hypo -methylation pattern were retained as shown with the normalized, un-adjusted z-scores (Fig. 3) in the associated patient groups while the clusters at gender levels were more scattered in normal groups compared to that from the un-adjusted z-scores (Additional file 1: Fig. S6 and S7). This result suggested that the effects that gender on population level clustering were effectively reduced.

### Statistical power evaluation with simulated data

In order to determine how the statistical power of our method might vary with the size of the control cohort used in our modeling, we developed a simulated dataset with a known ground truth hypermethylation effect representing a single probe. We implemented a statistical power analysis in order to show how our method using adjusted age and sex models for outlier detection improves performance over the base case of a model using unadjusted methylation values. We then demonstrate how the adjusted model used in conjunction with machine learning methods performs on real samples in our cohort.

**Fig. 5:**
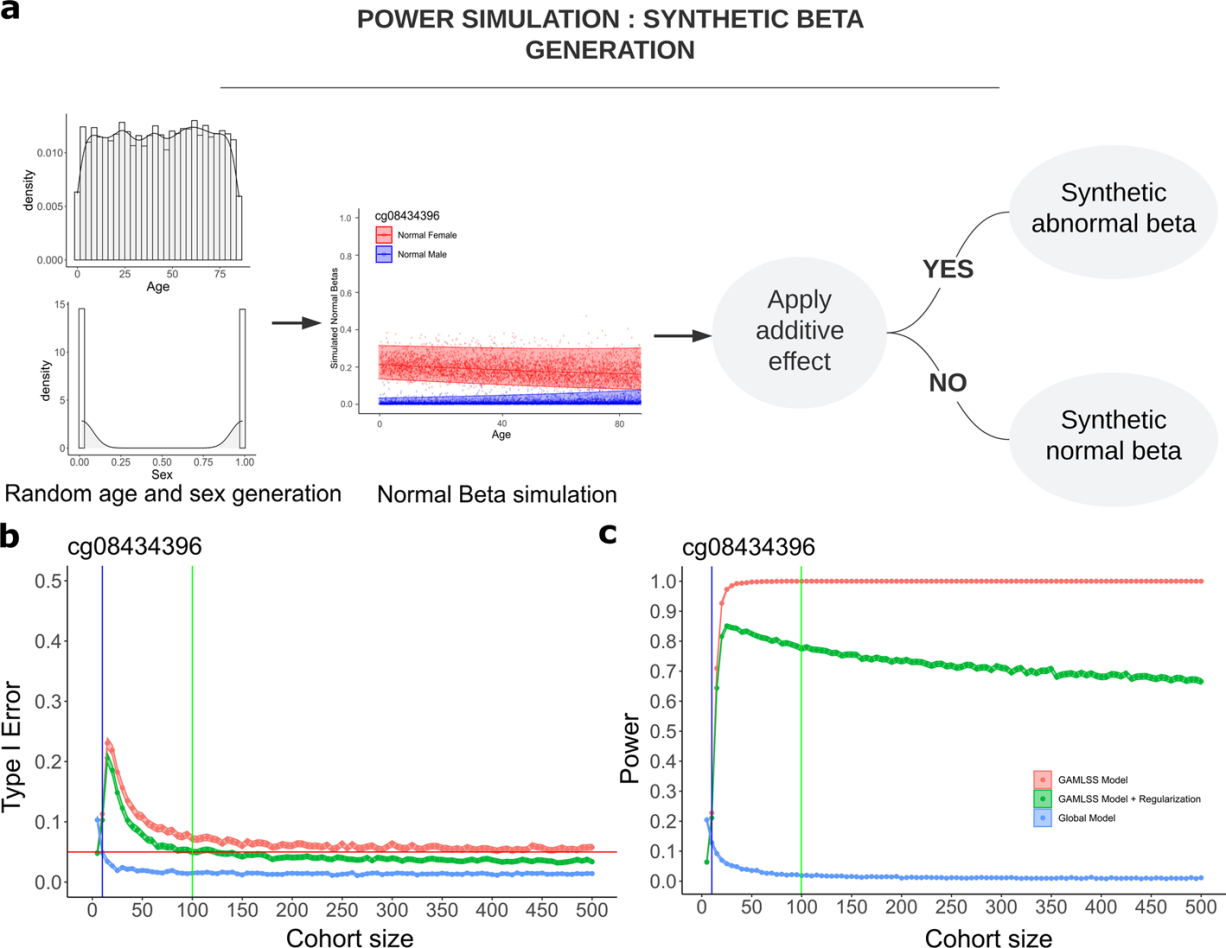
PowerAnalysis. (**a**) A diagram showing the workflow of power analysis based on simulation. (**b**) Type 1 error over cohort size for the 3 models, global, GAMLSS adjusted, and regularized. The red line indicates a type 1 error rate of 5 percent, which is considered a well-controlled error rate for this analysis. The blue and green lines show approximately where the model reaches the well-controlled type 1 error. (**c**) Power analysis over cohort size for the 3 models. The global model, while well-controlled at very small cohort sizes, has very low statistical power. The GAMLSS adjusted model, while having very high statistical power, requires a large cohort to control type 1 error. The regularized model controls for type 1 error at a cohort size smaller than that available to this study while still greatly improving the statistical power over the global model

We generate our synthetic methylation data by first fitting a Generalized Additive Model (GAM) to the control data (see Methods), then sampling from the distribution defined by the GAM fit for a single probe. This allows us to generate any number of synthetic data points while maintaining a reasonable approximation of the methylation patterns in the real data as captured by the chosen probe. For this analysis, we use our GAM for the probe cg08434396. To generate a single synthetic data point, we first randomly select a sex using equal probability male or female and an age using a uniform distribution from 0 to 85 years old (Fig. 5a). We then sample a “synthetic beta” value from the binomial distribution represented by the mu and sigma of the GAM for the age and sex combination that most closely matches that of this synthetic data point. After generating a synthetic cohort of size n, we fit a new GAM to this synthetic cohort to use as the basis of comparison for outlier detection in the power analysis. Finally, we generate a single additional synthetic data point to serve as a control and a second additional data point to serve as the synthetic hypermethylated abnormal by adding a constant to the simulated beta value. A constant of 0.3 was chosen for the effect size after initial exploration of possible effect sizes and probe models (Additional file 1: Fig. S8).

Three versions of the outlier detection are compared. First, an “unadjusted” model generates a z-score of the control and abnormal samples against the global mean and standard deviation of the entire synthetic control cohort. An “adjusted” model generates a z-score against the GAM for the synthetic control cohort. Finally, as a compromise between the first two approaches which is meant to reduce the impact of methylation outliers, a “regularized” model generates the z-score against the GAM for the synthetic control cohort after a regularization towards the global mean has been applied. A p-value for each version is also computed. We repeat the process of generating a synthetic cohort and the synthetic control and abnormal samples for 10,000 iterations at each cohort size from size 5 to 500 in steps of 5. The type 1 error rate and statistical power for these three models are shown in Figs. 5b, c. While the unadjusted model showed a well-controlled type 1 error of 0.05 at low cohort size, it suffered from a very low statistical power. The adjusted model achieved the highest statistical power but did not reach a well-controlled type 1 error rate in the range of cohort size simulated here. The regularized method achieved a well-controlled type 1 error at a cohort size of 100 coinciding with a statistical power of 0.775. This suggest that the cohort used in this study is exceeding the minimal cohort size for detecting outliers while controlling for type 1 error.

### Machine learning predicts disease class with high accuracy

Detection of individual probe outliers is an important aspect of clinically relevant methylation screening pipelines. However, manual review of outlier probes is only feasible on a small scale. In the case of SRS and other methylation disorders which present with global methylation patterns, we needed to develop our method to interrogate the combined effects of the global methylation levels of the approximately 850,000 probes in the EPIC array. We implemented a machine learning classification scheme as follows. We first split our cohort into an 80/20 train and test set. For the train set, we created the age and sex adjusted GAM as described above. We used this model to generate adjusted z-scores as defined above for both the train and the test set. As a means of reducing the number of potential features to examine in the downstream machine learning, we filtered the data removing probes rarely significantly differentially methylated in the cohort as described in the methods. We extracted 98 unique probes from our target region, and for the non-target probes that passed the previous filters we applied Uniform Manifold Approximation and Projection (UMAP) to reduce this feature set of 109,135 probes to 50 global features representing methylation patterns across the global methylation space save for the target region. We then combined the 98 probes and the 50 global features to generate a 148-feature dataset. Finally, we removed highly correlated features with a correlation cutoff of 0.9 from this set. In order to avoid data leakage, we selected probes and UMAP features using only the train set and applied these criteria to the test set as a separate step.

AutoGluon was used to train the classifier to predict disease class. AutoGluon employs an ensemble learning method using several individual models including ’LightGBM’, ’XGBoost’, ’Random Forest’, ’CatBoost’, a feedforward ’Neural Net’ implemented with MXnet Gluon, and ’K Nearest Neighbors’ [27]. A weighted ensemble model is automatically generated from the stacked input models. We employed a 5-fold cross-validation within the training set by setting the AutoGluon parameter numBagFolds = 5 and report the cross-validation accuracy of AutoGluon’s component models (Additional file 1: Fig. S9). The cross-validation accuracy of the weighted ensemble model was 0.978355. After cross-validation, the model was used to predict classes for the test set. The model correctly classified all the 29 control samples in the test set as normal with an average probability of 0.913740 (Additional file 1: Table S3). The samples with imprinting disorders labeled as BWS2, AS2, PWS1, PWS2 and FXS were also correctly classified (Table 2). Two misclassified samples included a BWS1 which was misclassified as BWS2 and a SRS1 which was misclassified as normal (Table 2, shown in bold). The combined accuracy of prediction by this model was 0.9615 (Binomial 95% Confidence Interval 0.868$-$0.995). A classifier trained from the features engineered using the same method from unadjusted z-scores yielded an overall accuracy of 0.90 with a binomial 95% confidence interval from 0.79 to 0.97, which is lower in accuracy and higher in variability. These results suggested that the model we trained using the methylation data with sex and age adjustment could successfully discriminate abnormality from normality and with a high accuracy of predicting specific disease status.

## Discussion

In this study, we performed a whole genome methylation study of a cohort with 283 patients using the Illumina MethylationEPIC BeadChip [18]. The control cohort included in this study is, to our best knowledge, one of the largest reference methylomes for whole blood samples from ages 0 through 81 years old including both male and female. A key limitation in current clinical detection of methylation abnormalities is MS-MLPA tests only detecting a handful of well-characterized methylation sites. In order to detect methylation abnormalities which are present more variably across the genome, it is necessary to take genomic methylation patterns into account. As more methylation sites are utilized, the need exists to account for known patterns such as methylation level changes over age and sex at certain probe locations. Thus, we developed a general additive model implemented in the GAMLSS package [28] to correct age and sex effects on methylation levels and to enable precise outlier detection for any given probe in a particular male or female patient at a specific age.

Simulating cohorts with different numbers of individuals drawn from a population of uniformly distributed age and sex for a probe with a hypermethylation effect size of 0.3 revealed that a cohort of 100 samples could effectively achieve statistical power of 0.775 with type 1 error controlled at 0.05. Given our cohort size included 149 controls, our model likely achieved a well-controlled type 1 error rate while also improving the statistical power over a base model using a globally derived reference range. While our method was able to achieve high classification accuracy, we recognize there were two misclassified samples in the results. One SRS1 sample was misclassified as normal with a predication probability of 0.433498. We observed that the predication probability of this sample being classified correctly as SRS1 is 0.413866, which is slightly less than that of the incorrectly called class. We consider this as a ’near miss’ in the sense that the top two prediction probabilities for this sample were both relatively low scores and were relatively similar scores, which seems to indicate that the classifier did not have strong confidence in one call over the other. In a clinical setting, this would still be a useful data point which could indicate a need for further investigation. The other error was a BWS1 sample which was misclassified as BWS2 with a prediction probability of 0.812139. The second highest prediction probability for this sample was at 0.118237 for the correct class of BWS1. In light of the fact that the methylation loci know to be associated with BWS1 and BWS2 overlap, we consider this misclassification another ’near-miss’. Again, in a clinical setting, this data point would still be useful and may indicate a need for samples classified positively for any disorder to be confirmed using an orthogonal method.

This study demonstrates the potential for methylation disorder classification using a combination of targeted probes and global methylation features generated using UMAP for dimensionality reduction. UMAP and other dimensionality reduction tools such as Principal Component Analysis (PCA) or t-Distributed Stochastic Neighbor Embedding (t-SNE) [29] are frequently used to reduce high-dimensional -omics data to lower dimensions for visualization or machine learning applications [30, 31]. A recent study has demonstrated that high dimensional data can become distorted relative to the original space when reduced to very few dimensions [32]. While this study found that 50 dimensions was sufficient to supplement the targeted features and lead to strong classifier performance in this dataset, it did not attempt to show that 50 dimensions was optimal or that it would necessarily generalize to other datasets.

It is also important to consider that the majority of samples in this study were taken from young patients from the Mayo Clinic, and as such these results may not be representative of outside populations. In particular, it would be of great interest in a future study to include more samples from older patients with confirmed disorders. The low availability of such samples is likely due to higher mortality in older adults with congenital disease and participant recruitment readiness [33, 34, 34–36]. The majority of confirmed abnormal samples in this cohort were taken from patients younger than 1 year old in both male and female for each disorder except FXS cases which occur only in males (Fig. 2a, b). We also acknowledge a sex bias in our abnormal samples, which was a result of there not being enough samples available for each disorder class for both sexes. The ratio for our cohort was in favor of males for BWS (male-to-female ratio: 1.24), AS2 (male–female-ratio: 2.0) and PWS1 (male–female-ratio: 1.43), while being in favor of females for SRS1 (male-to-female ratio: 0.79). The samples without any confirmatory clinical diagnosis of congenital disease, labeled as normal, are evenly distribution across all age groups in both male and female samples with a male-to-female ratio of 1.01 (75/74). The broad sampling of normal cases across ages and sex provided sufficient data for standardizing a normal methylation range for methylation outlier identification.

## Conclusion

The development of a single genome-wide methylation screening test for a large variety of methylation disorders remains an important goal for clinical testing. Age and sex effects are widely known to exist in the DNA methylome and are known to vary in significance between probes. Previous works have sought to account for age and/or sex effects in DNA methylation analysis. This work demonstrates a novel method to adjust for age and sex effects using a stratified cohort of normal samples to build a series of generalized models which are then applied for per-probe outlier detection. We show how adjusting for age and sex can improve the statistical power of outlier detection particularly in the subset of probes that include a strong age or sex effect. We further demonstrate in a novel cohort of 283 samples how our method can be used to generate both targeted and global methylation features which can be used in machine learning classification to achieve high accuracy in classifying several methylation disorders.

The results of this study are promising and could be useful for future development of a clinically validated set of features which can predict methylation disorders including diseases which are defined by global methylation patterns. Our method was able to demonstrate a significant increase in power over the baseline method at our cohort size while still controlling for type 1 error rates. The adjusted model achieved a higher accuracy than a similar model generated without age and sex adjustment, though the difference was not statistically significant as the adjusted model’s performance was slightly within the unadjusted model’s 95% CI. A larger cohort in the test data might feasibly be able to demonstrate a statistically significant improvement in the adjusted model over the unadjusted model. Additionally, with a significantly large control cohort, it may be possible to control type 1 error rates without regulating the adjusted model toward the global mean which may further improve the statistical power of these methods.

## Materials and methods

### Whole genome methylation assay and data processing

Residual DNA from each patient was clinically isolated from whole blood. Approximately 1250 ng of each patient DNA was bisulfite converted using the Bisulphite treatment was performed using EZ DNA Methylation Spin Column kit (Zymo) per manufacturer’s instructions. Two rounds of bisulfite conversion were performed on each sample to ensure thorough bisulfite conversion of methylated cytosines. The DNA samples passed a DNA quantification QC check and then proceeded for processing onto the Infinium MethylationEPIC 850k (EPIC) arrays. Each 96-well plate of samples were processed into 12 arrays yielding one batch of samples. A total of six batches were processed into EPIC arrays [18]. The processed arrays were scanned on an iScan to obtain methylation results. A total of 576 samples were run (including controls). The cohort used in this study excludes those samples with suspected imprinting issues (based on chart review), those with equivocal MS-MLPA results, and non-patient control samples, resulting in the final cohort of 283 samples.

### Adjusting for age and sex using GAMLSS

We implemented the age and sex adjustment as follows. We first performed ChAMP normalization (see above) which resulted in 746,834 probes retained after quality control and filtering. After the 80/20 split, we then separated the controls from the abnormals in the train set. All beta values were logit transformed. We define an unadjusted z-score for a single logit-transformed methylation beta value against the global mean (globalMu) and the global standard deviation (globalSigma) of the normal cohort using the formula:
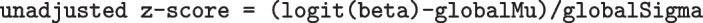


For each individual probe, we fit a GAM to the the logit transformed beta values using the R package GAMLSS command:
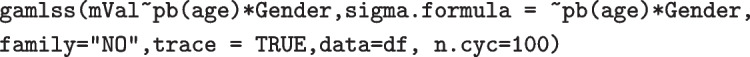


resulting in a GAM for each probe. We converted the GAM into discrete age and sex combinations as the original model was prohibitively computationally expensive to store and use. Ages were defined in one day increments from 0 to 365 days old, then from 1 month to 24 months in 1 month increments, and finally from age 3 to 85 in 1 year increments. The GAMLSS method “predictAll” was used to generate a mu and sigma for each of the discretized age and sex combinations. A z-score can be computed for any sample by comparing to the mu and sigma values of the closest discrete age and sex values to the sample. We define an adjusted z-score for a single logit-transformed methylation beta value against the GAM model using the formula:
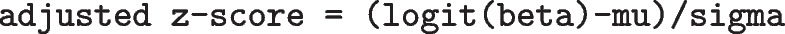


A regularization was applied to the adjusted z-score by applying the following transformation:
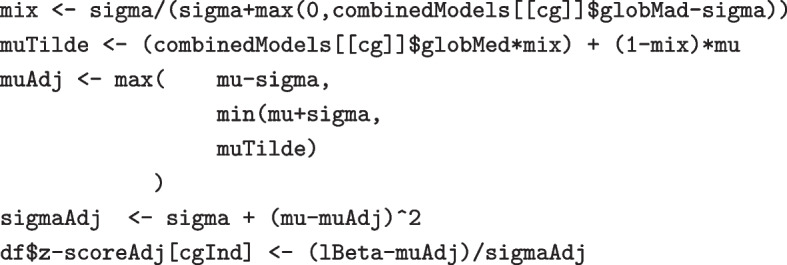


### Feature selection and classify model training

For classification, an original set of features composed of 98 [15] unique probes passed the z-score filter with known annotation for clinical utility and 50 global features extracted by UMAP from the remaining probes were used for further feature filtering [37]. To remove similarity and redundancy of the features, a pairwise correlation analysis between the original features were conducted to examine the similarities using the “findCorrelation” function in caret R-package (v6.0-90) [38]. For the pairs with correlation coefficient higher than 0.9, one of the pair with the largest mean absolute correlation was removed from the feature matrix. The final feature set, after quality filters and correlation filters, reduced the combined 148 targeted and global features to 70 features for modeling. The train/test split was conducted in an 80/20 ratio using the “createDataPartition” function in caret R-package [38]. Models for classification were trained and evaluated for the accuracy using AutoGluon (v0.3.0) [27]

**Table 1:** Number of samples included in each of the categories for the train and test cohorts

	Normal	BWS1	BWS2	SRS1	AS2	PWS1	PWS2	FXS
Train set	120	15	16	28	15	14	15	8
Test set	29	3	3	6	3	3	3	2
Total	149	18	19	34	18	17	18	10

**Table 2:** Confusion matrix, precision, recall, and F1 Score of AutoGluon prediction. Misclassified samples are shown in bold

	Normal	BWS1	BWS2	SRS1	AS2	PWS1	PWS2	FXS	Precision	Recall	F1 Score
Normal	29	0	0	**1**	0	0	0	0	0.97	1.00	0.98
BWS1	0	2	0	0	0	0	0	0	1.00	0.67	0.80
BWS2	0	**1**	3	0	0	0	0	0	0.75	1.00	0.86
SRS1	0	0	0	5	0	0	0	0	1.00	0.83	0.91
AS2	0	0	0	0	3	0	0	0	1.00	1.00	1.00
PWS1	0	0	0	0	0	3	0	0	1.00	1.00	1.00
PWS2	0	0	0	0	0	0	3	0	1.00	1.00	1.00
FXS	0	0	0	0	0	0	0	2	1.00	1.00	1.00

The items in bold in Table 2 are samples that were misclassified by the model

### Supplementary Information

**Additional file 1**. Supplemental figures and tables.

## Data Availability

The data and code used in this study are available upon reasonable request.
