# Supplementary material for: A supervised learning method for classifying methylation disorders
Source: BMC Bioinformatics. 2024 Feb 12;25:66. doi: 10.1186/s12859-024-05673-1 (PMC10863277; doi:10.1186/s12859-024-05673-1)
Supplement: Supplementary file 1 — Additional file 1. Supplemental figures and tables. [file 12859_2024_5673_MOESM1_ESM.pdf]

Additional file 1

A supervised learning method for classifying methylation disorders

Jesse R. Walsh[1,#], Guangchao Sun[1,#], Jagadheshwar Balan[1], Jayson Hardcastle[1], Jason Vollenweider[1], Calvin Jerde[1], Kandelaria Rumilla[1], Christy Koellner[1], Alaa Koleilat[2], Linda Hasadsri[1], Benjamin Kipp[1], Garrett Jenkinson[1] and Eric Klee[1*]

Author Affiliations

1 Mayo Clinic, Rochester, MN, United States
2 Department of Molecular and Medical Genetics, Oregon Health & Science University, Portland, OR, United States
# Equal contributors
* Corresponding author

Legend

Fig. S1. Principal components of SRS.

Fig. S2. Clusters.

Fig. S3. Identify the principal component that capture sex difference in methylation.

Fig. S4. Identify the principal component that capture age difference in methylation.

Fig. S5. Beta plots for select probes.

Fig. S6. Clustering based on adjusted z-scores of probes known to be involved in the abnormalities included in this study.

Fig. S7. Scatteredness.

Fig. S8. Power analysis simulations exploring effect size over and different probes.

Fig. S9. 5-fold cross-validation accuracy of AutoGluon's component models.

Table S1. List of Relevant Probes and Disease Associations.

Table S2. Accumulative variance explained by principal components calculated from the 109,131 above-background-noise probes among 283 samples included in the studied cohort.

Table S3. Possibility of each samples in test sets being predicted as each of the classes by the classifier trained in this study after adjustment and calibration.

Supplementary Figures.

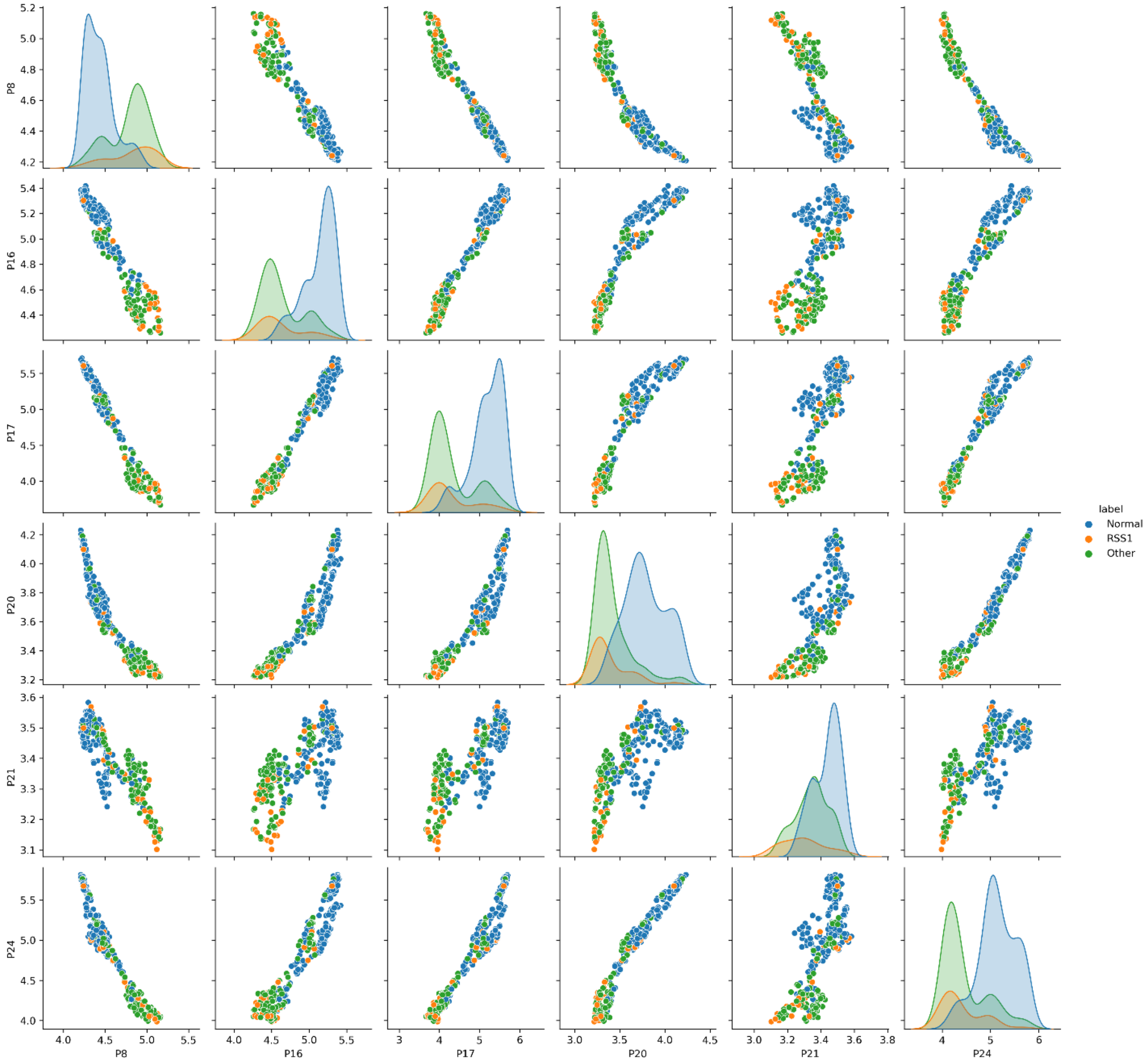

Fig. S1. Principal components of SRS. Global features represented by selected UMAP dimensions were not able to distinguish SRS patients from the cohort.

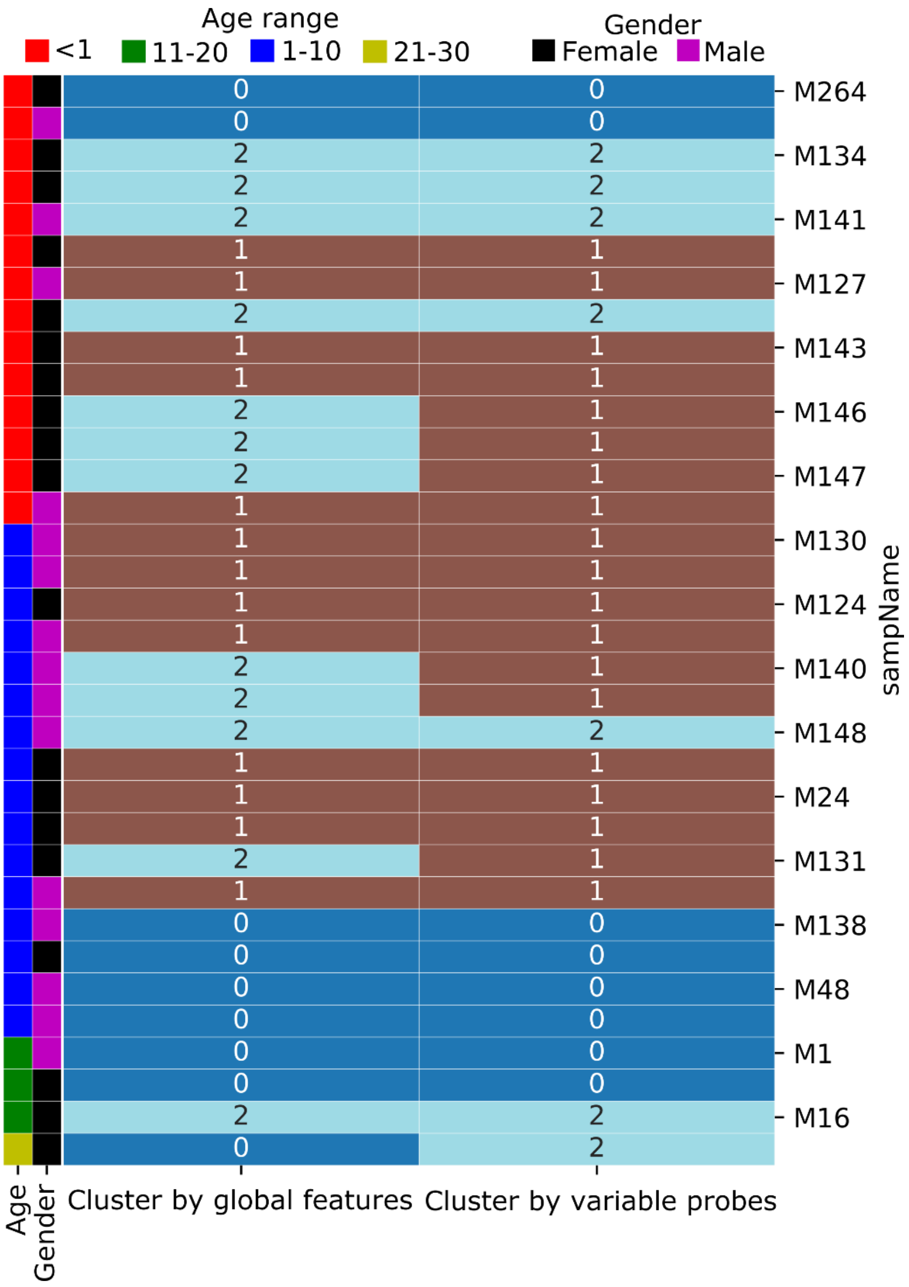

Fig. S2. Clusters. K-Mean clustering based on highly variable probes or 30 UMAP dimensions extracted from these probes did not put SRS patients in a single cluster. The entire cohort was forced to be clustered into 3 groups based on probes of which the methylation level showing deviation higher than 1.5 or UMAP dimensions extracted from these highly variable probes. Clustering results for SRS patients were shown in the heatmap.

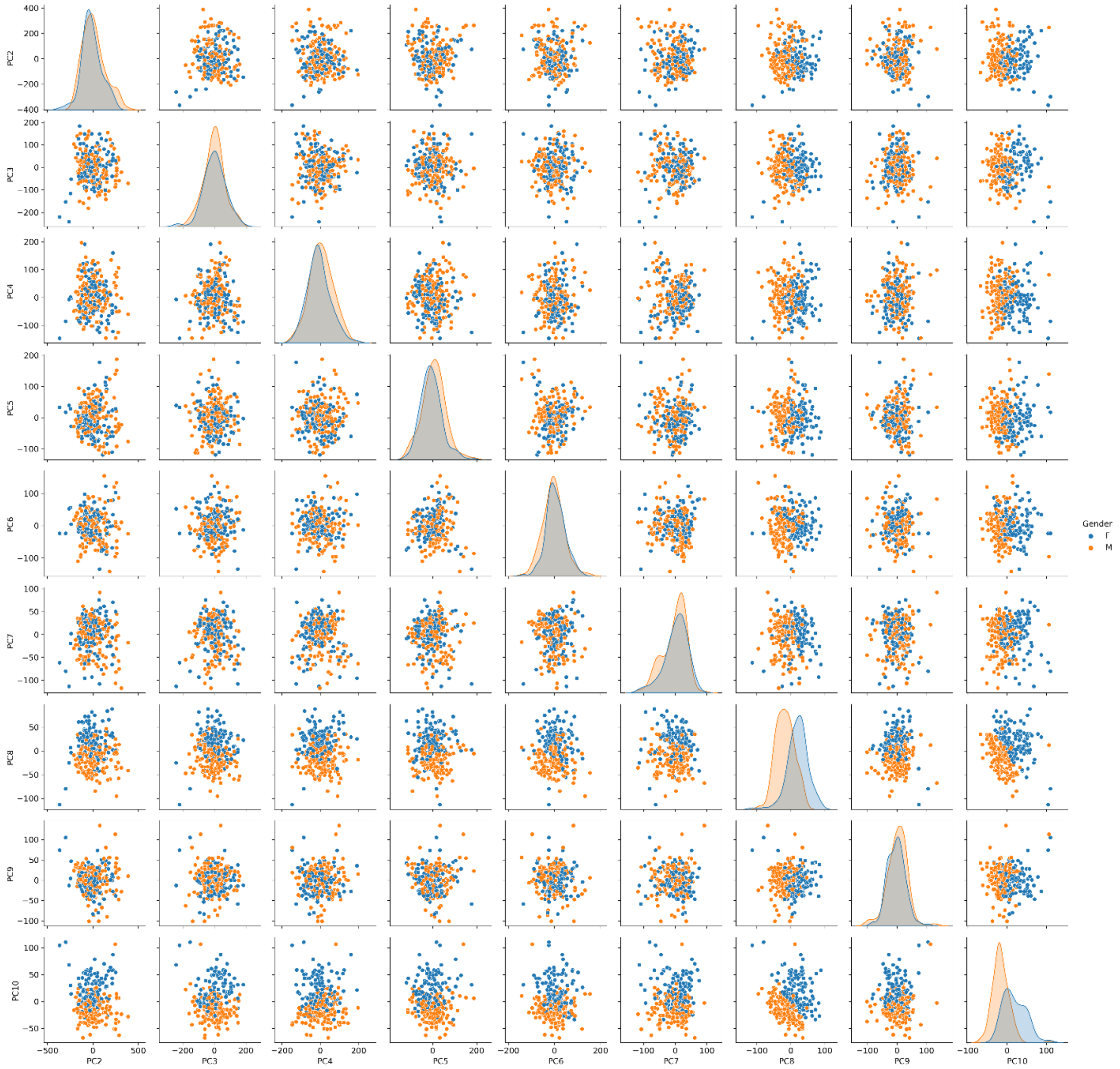

Figure S3. Sex difference in methylation. Pair plot for 10 principal components are calculated based on normalized beta values of 109,131 above-background-noise probes among 283 samples included in the studied cohort. Sex was color coded.

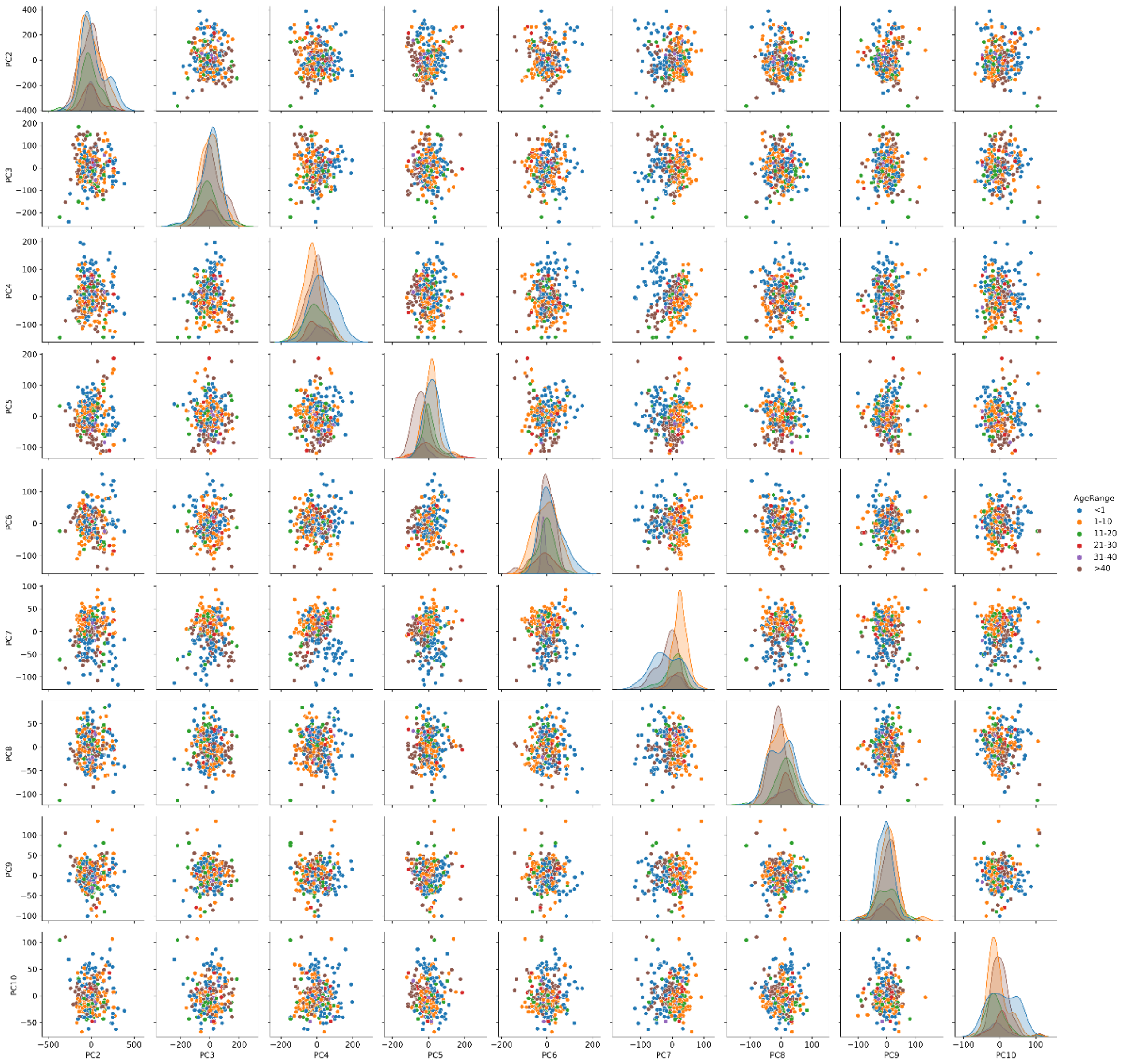

Figure S4. Age difference in methylation. Pair plot for 10 principal components are calculated based on normalized beta values of 109,131 above-background-noise probes among 283 samples included in the studied cohort. Sex was color coded. Age group was color coded.

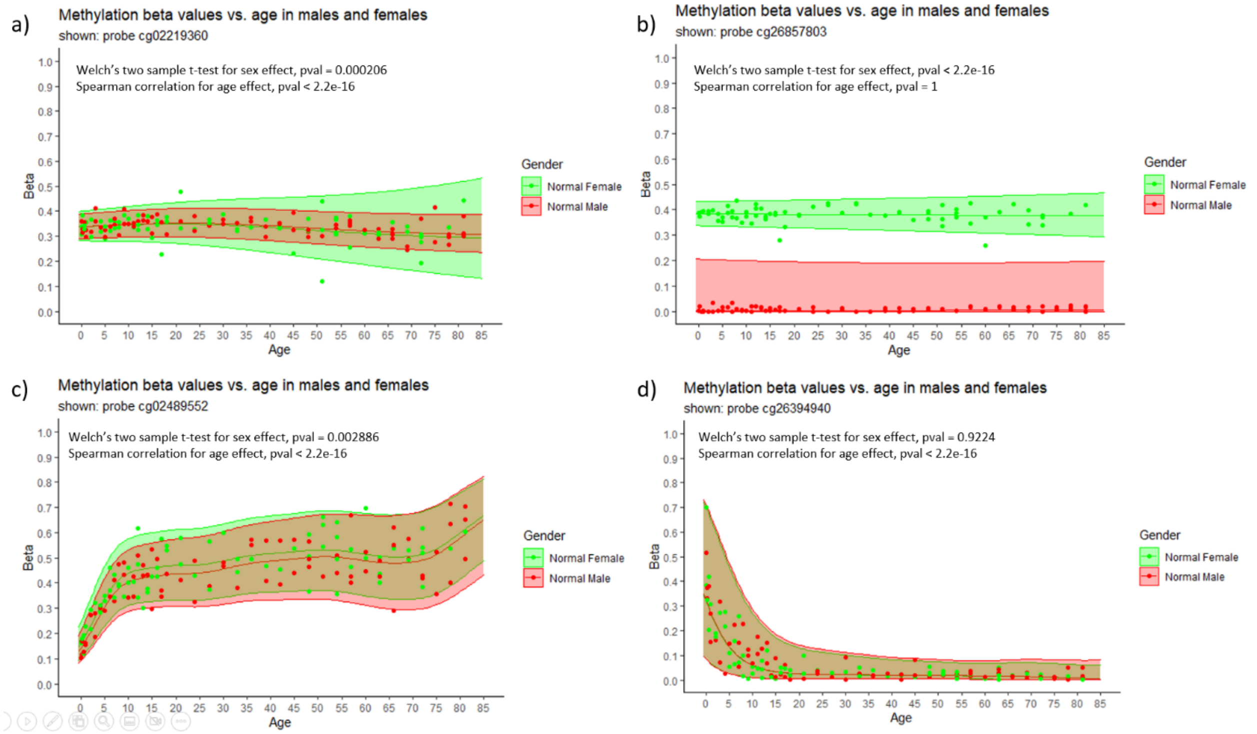

Fig. S5. Beta plots for select probes. Probe beta values plotted against subject age in years for normal (apparently healthy) samples. Females are shown in green, males in red, with the mean line in the center and shading for two standard deviations from the mean. Four select probes are shown as examples of age and sex effects. For each probe, a Welch's two sample t-test is used to compare the male and female groups testing for significant differences between male and female sexes. Also shown is the Spearman correlation comparing the combined male and female samples against age to test for statistically significant correlation between beta values and age. Probes in (a) and (b) show relatively low age effect, although the age effect was statistically significant (pval < 0.05) in (a). In (b), a strong sex effect can be observed. Probes in (c) and (d) are represented in the list of probes published by Horvath's clock (Horvath 2013) as probes shown to have strong age effects.

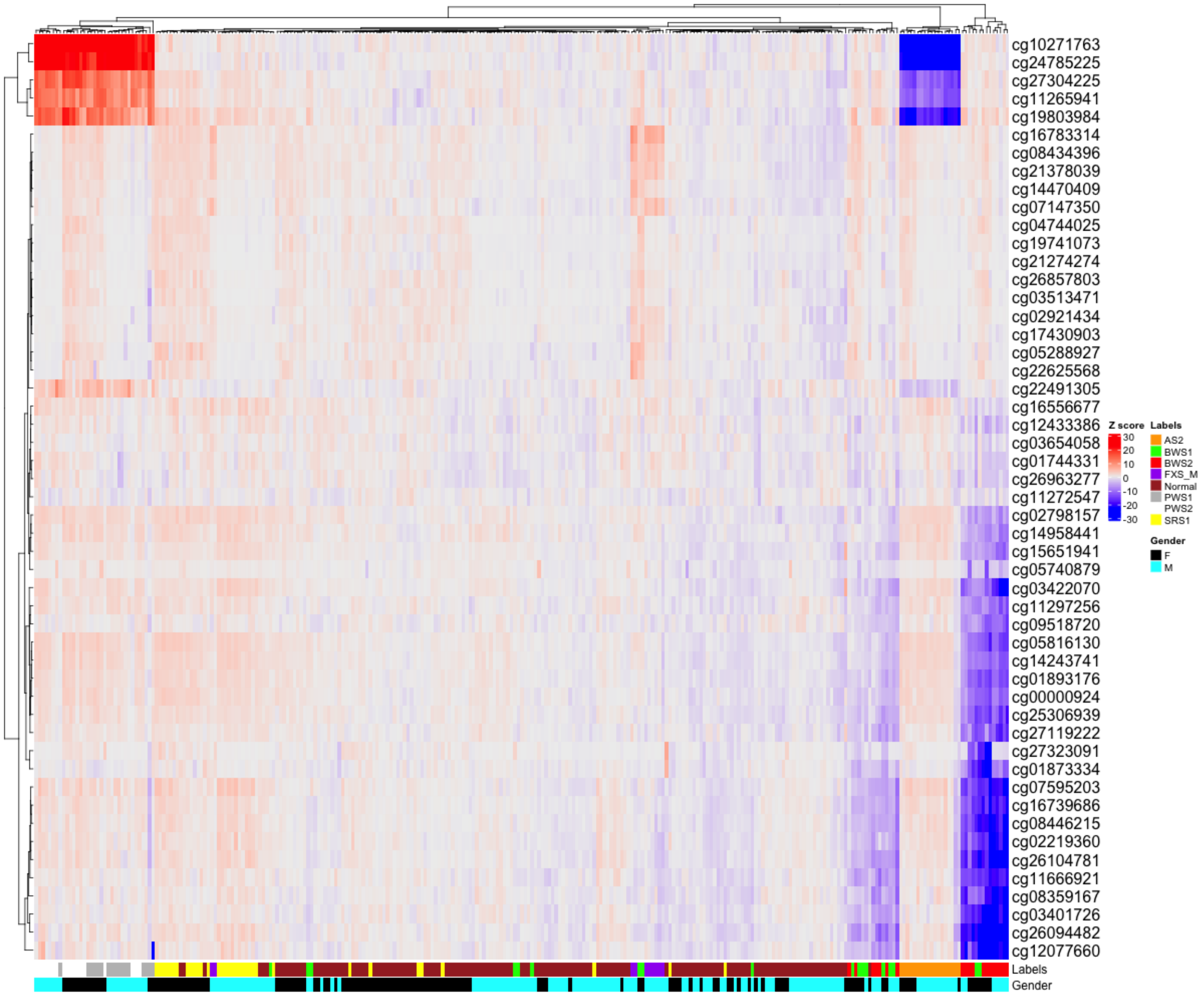

Figure S6. Clustering based on adjusted z-scores. z-scores were calculated based on the probe beta values of the samples and adjusted for age and sex used by the model developed in this study. The samples were clustered using k-means method.

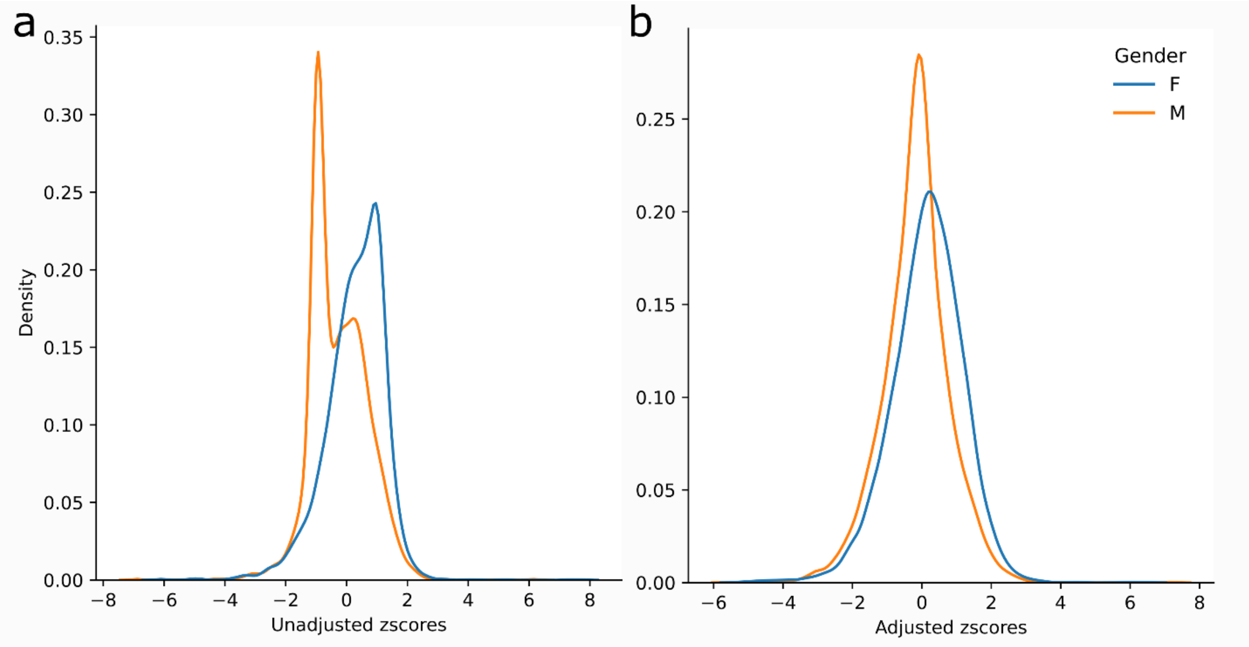

Fig. S7. Scatteredness. The distribution of z-scores calculated based on raw (a) or sex/age adjusted (b) methylation levels (beta) of target probes in male and female normal samples.

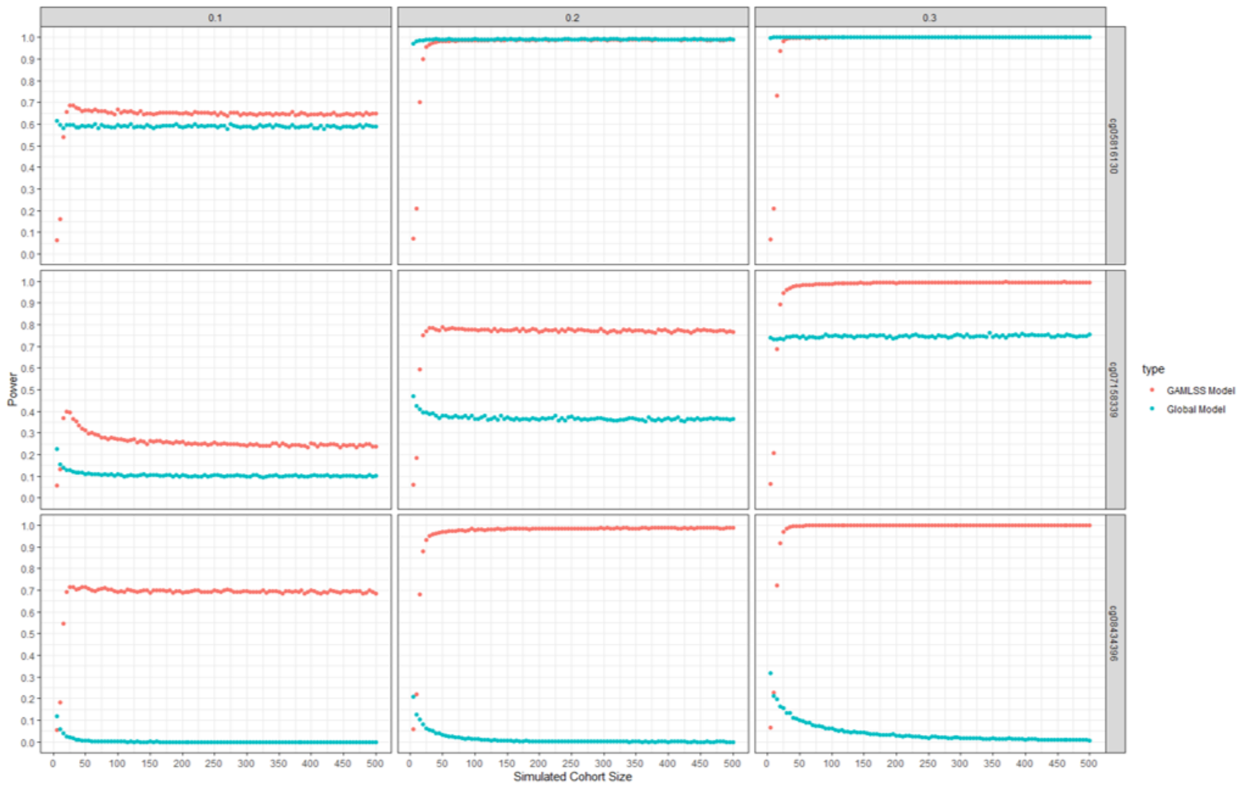

Fig. S8. Power analysis simulations exploring effect size and different probes. The age and sex adjusted GAMLSS model is shown in red, while the unadjusted global mean model is in blue. The simulation was performed for the effect sizes 0.1, 0.2, and 0.3 (going from left to right columns) for three different probes. Probes cg05816130, cg07158339, and cg08434396 were arbitrarily chosen by visual inspection of betas to represent weak, medium, and strong effects from age and/or sex (from top row to bottom). Probes with stronger age and/or sex effects show greater difference between the power of the adjusted and unadjusted model, and show very little difference when there is almost no age and/or sex effect for that probe.

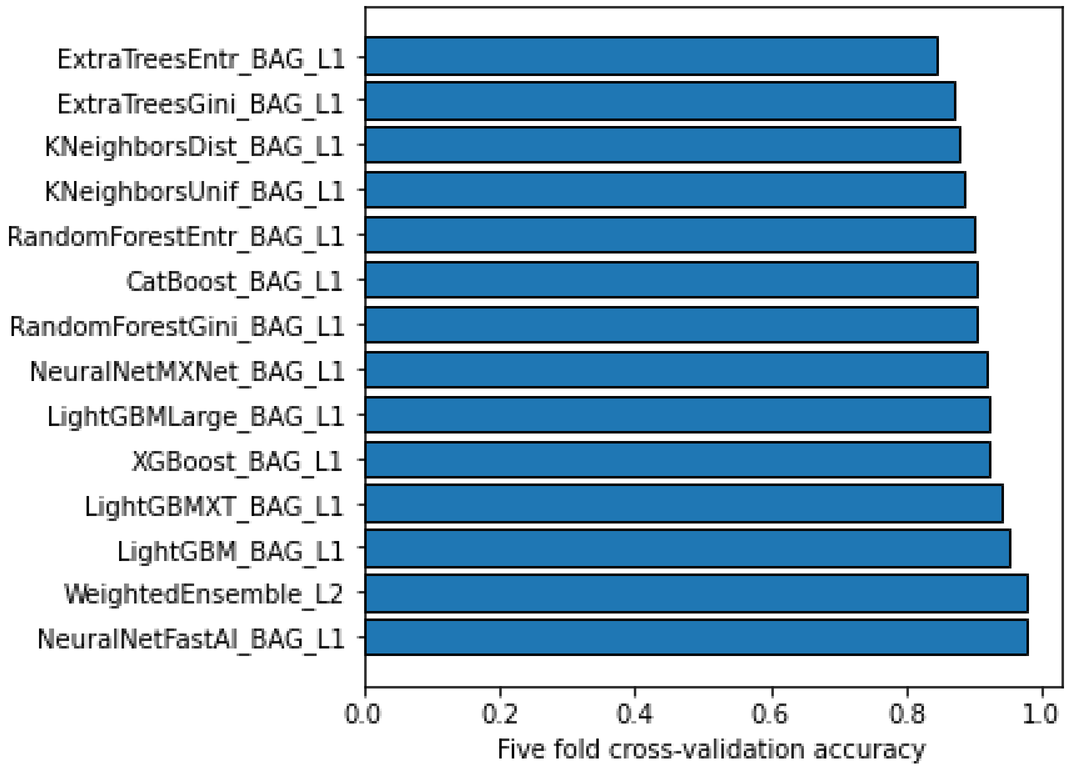

Figure S9. AutoGluon 5-fold cross-validation accuracies. The validation accuracy of models used for ensemble learning.

Supplementary Tables.

Table S1. List of Relevant Probes and Disease Associations

Probe	Chromosome	Position	Strand	Annotation
cg17769238	chr11	2019568	+	04_H19DMR__BWS
cg02694715	chr11	2019730	-	05_H19DMR__BWS
cg11735853	chr11	2019732	+	05_H19DMR__BWS
cg01977486	chr11	2019736	+	05_H19DMR__BWS
cg27119222	chr11	2720229	+	KCNQ1OT1_locus_Hypo_is_Beckwith_Wiedeman
cg00000924	chr11	2720463	+	KCNQ1OT1_locus_Hypo_is_Beckwith_Wiedeman
cg11297256	chr11	2720810	-	KCNQ1OT1_locus_Hypo_is_Beckwith_Wiedeman
cg12077660	chr11	2721243	+	KCNQ1OT1_locus_Hypo_is_Beckwith_Wiedeman
cg03401726	chr11	2721248	+	KCNQ1OT1_locus_Hypo_is_Beckwith_Wiedeman
cg16739686	chr11	2721336	-	KCNQ1OT1_locus_Hypo_is_Beckwith_Wiedeman
cg08359167	chr11	2721351	+	KCNQ1OT1_locus_Hypo_is_Beckwith_Wiedeman
cg08446215	chr11	2721366	-	KCNQ1OT1_locus_Hypo_is_Beckwith_Wiedeman
cg26104781	chr11	2721383	-	KCNQ1OT1_locus_Hypo_is_Beckwith_Wiedeman
cg02219360	chr11	2721409	-	KCNQ1OT1_locus_Hypo_is_Beckwith_Wiedeman;20_KvDMR__BWS
cg07595203	chr11	2721480	-	KCNQ1OT1_locus_Hypo_is_Beckwith_Wiedeman
cg09518720	chr11	2721591	-	KCNQ1OT1_locus_Hypo_is_Beckwith_Wiedeman
cg14958441	chr11	2721610	-	KCNQ1OT1_locus_Hypo_is_Beckwith_Wiedeman
cg27323091	chr11	2721619	-	KCNQ1OT1_locus_Hypo_is_Beckwith_Wiedeman
cg01873334	chr11	2721632	-	KCNQ1OT1_locus_Hypo_is_Beckwith_Wiedeman
cg05816130	chr11	2721799	+	KCNQ1OT1_locus_Hypo_is_Beckwith_Wiedeman
cg14243741	chr11	2721817	+	KCNQ1OT1_locus_Hypo_is_Beckwith_Wiedeman
cg05740879	chr11	2721866	-	KCNQ1OT1_locus_Hypo_is_Beckwith_Wiedeman
cg01893176	chr11	2721952	-	KCNQ1OT1_locus_Hypo_is_Beckwith_Wiedeman
cg11666921	chr11	2722062	+	KCNQ1OT1_locus_Hypo_is_Beckwith_Wiedeman
cg26094482	chr11	2722073	+	KCNQ1OT1_locus_Hypo_is_Beckwith_Wiedeman
cg03422070	chr11	2722082	+	KCNQ1OT1_locus_Hypo_is_Beckwith_Wiedeman
cg15651941	chr11	2722084	+	KCNQ1OT1_locus_Hypo_is_Beckwith_Wiedeman
cg25306939	chr11	2722086	+	KCNQ1OT1_locus_Hypo_is_Beckwith_Wiedeman
cg06288089	chr11	2722119	+	KCNQ1OT1_locus_Hypo_is_Beckwith_Wiedeman
cg02798157	chr11	2722195	+	KCNQ1OT1_locus_Hypo_is_Beckwith_Wiedeman
cg12433386	chr11	2722340	+	KCNQ1OT1_locus_Hypo_is_Beckwith_Wiedeman
cg01744331	chr11	2722358	+	KCNQ1OT1_locus_Hypo_is_Beckwith_Wiedeman
cg16556677	chr11	2722401	-	KCNQ1OT1_locus_Hypo_is_Beckwith_Wiedeman
cg26963277	chr11	2722407	-	KCNQ1OT1_locus_Hypo_is_Beckwith_Wiedeman
cg11272547	chr11	2722440	-	KCNQ1OT1_locus_Hypo_is_Beckwith_Wiedeman
cg03654058	chr11	2722539	-	KCNQ1OT1_locus_Hypo_is_Beckwith_Wiedeman
cg20069718	chr12	50898249	+	DIP2B_promoter_hyper_is_CGG_repeatExpansion_FRA12A_mentalRetardation

cg25874642	chr12	50898251	+	DIP2B_promoter_hyper_is_CGG_repeatExpansion_FRA12A_mentalRetardation
cg03907575	chr12	50898411	+	DIP2B_promoter_hyper_is_CGG_repeatExpansion_FRA12A_mentalRetardation
cg20014596	chr12	50898483	+	DIP2B_promoter_hyper_is_CGG_repeatExpansion_FRA12A_mentalRetardation
cg18650092	chr12	50898549	+	DIP2B_promoter_hyper_is_CGG_repeatExpansion_FRA12A_mentalRetardation
cg12980886	chr12	50898622	-	DIP2B_promoter_hyper_is_CGG_repeatExpansion_FRA12A_mentalRetardation
cg05827208	chr12	50898684	-	DIP2B_promoter_hyper_is_CGG_repeatExpansion_FRA12A_mentalRetardation
cg10699967	chr12	50898744	-	DIP2B_promoter_hyper_is_CGG_repeatExpansion_FRA12A_mentalRetardation
cg01096478	chr12	50898751	-	DIP2B_promoter_hyper_is_CGG_repeatExpansion_FRA12A_mentalRetardation
cg15661672	chr12	50898761	-	DIP2B_promoter_hyper_is_CGG_repeatExpansion_FRA12A_mentalRetardation
cg10061107	chr12	50898763	-	DIP2B_promoter_hyper_is_CGG_repeatExpansion_FRA12A_mentalRetardation
cg21598490	chr12	50898946	-	DIP2B_promoter_hyper_is_CGG_repeatExpansion_FRA12A_mentalRetardation
cg23142731	chr12	50899018	-	DIP2B_promoter_hyper_is_CGG_repeatExpansion_FRA12A_mentalRetardation
cg00665405	chr12	50899548	+	DIP2B_promoter_hyper_is_CGG_repeatExpansion_FRA12A_mentalRetardation
cg26088746	chr12	50899895	-	DIP2B_promoter_hyper_is_CGG_repeatExpansion_FRA12A_mentalRetardation
cg00764369	chr14	101290556	-	MEG3_promoter_imprinted_region
cg10515315	chr14	101290717	+	MEG3_promoter_imprinted_region
cg15991553	chr14	101290867	+	MEG3_promoter_imprinted_region
cg23870378	chr14	101291068	+	MEG3_promoter_imprinted_region
cg14034270	chr14	101291100	+	MEG3_promoter_imprinted_region
cg16567044	chr14	101291136	-	MEG3_promoter_imprinted_region
cg10540302	chr14	101291180	-	MEG3_promoter_imprinted_region
cg07501539	chr15	23809945	+	MKRN3_promoter_imprinted_region
cg09437135	chr15	23810163	+	MKRN3_promoter_imprinted_region
cg20769842	chr15	23810238	+	MKRN3_promoter_imprinted_region
cg16131766	chr15	23810280	-	MKRN3_promoter_imprinted_region
cg20792895	chr15	23810334	+	MKRN3_promoter_imprinted_region
cg05952543	chr15	23810378	+	MKRN3_promoter_imprinted_region
cg27046052	chr15	23810652	+	MKRN3_promoter_imprinted_region
cg03969797	chr15	23810664	+	MKRN3_promoter_imprinted_region
cg00215587	chr15	23810843	+	MKRN3_promoter_imprinted_region
cg13096454	chr15	23810849	+	MKRN3_promoter_imprinted_region
cg11100640	chr15	23810861	+	MKRN3_promoter_imprinted_region
cg23234999	chr15	23811205	+	MKRN3_promoter_imprinted_region
cg13415551	chr15	23811566	-	MKRN3_promoter_imprinted_region
cg20525221	chr15	23811572	-	MKRN3_promoter_imprinted_region
cg07760796	chr15	23812334	+	MKRN3_promoter_imprinted_region
cg22491305	chr15	25068564	-	SNRPN_SNURF_locus_Hyper_is_Prader_Willi_and_Hypo_is_Angelman
cg11265941	chr15	25068738	-	SNRPN_SNURF_locus_Hyper_is_Prader_Willi_and_Hypo_is_Angelman
cg11826663	chr15	25068754	+	SNRPN_SNURF_locus_Hyper_is_Prader_Willi_and_Hypo_is_Angelman
cg24785225	chr15	25068757	+	SNRPN_SNURF_locus_Hyper_is_Prader_Willi_and_Hypo_is_Angelman
cg10271763	chr15	25068763	+	SNRPN_SNURF_locus_Hyper_is_Prader_Willi_and_Hypo_is_Angelman

cg27304225	chr15	25068769	+	SNRPN_SNURF_locus_Hyper_is_Prader_Willi_and_Hypo_is_Angelman
cg19803984	chr15	25068790	+	SNRPN_SNURF_locus_Hyper_is_Prader_Willi_and_Hypo_is_Angelman
cg16744228	chr15	25200113	-	P14-PW-SRO_CpG_PWAS
cg17916021	chr15	25200133	-	P14-PW-SRO_CpG_PWAS
cg04072648	chr15	25200145	-	P14-PW-SRO_CpG_PWAS
cg02125271	chr15	25200406	-	P15-PW-SRO_CpG_PWAS
cg07147350	chrX	146992908	+	FMR1_promoter_repeat_locus_Hyper_in_male_is_FragileX
cg22625568	chrX	146993010	+	FMR1_promoter_repeat_locus_Hyper_in_male_is_FragileX
cg05288927	chrX	146993092	+	FMR1_promoter_repeat_locus_Hyper_in_male_is_FragileX
cg14470409	chrX	146993125	+	FMR1_promoter_repeat_locus_Hyper_in_male_is_FragileX
cg04744025	chrX	146993175	+	FMR1_promoter_repeat_locus_Hyper_in_male_is_FragileX
cg17430903	chrX	146993420	-	FMR1_promoter_repeat_locus_Hyper_in_male_is_FragileX
cg02921434	chrX	146993433	-	FMR1_promoter_repeat_locus_Hyper_in_male_is_FragileX
cg26857803	chrX	146993447	-	FMR1_promoter_repeat_locus_Hyper_in_male_is_FragileX
cg03513471	chrX	146993485	-	FMR1_promoter_repeat_locus_Hyper_in_male_is_FragileX
cg16783314	chrX	146993722	+	FMR1_promoter_repeat_locus_Hyper_in_male_is_FragileX
cg08434396	chrX	146993778	-	FMR1_promoter_repeat_locus_Hyper_in_male_is_FragileX
cg21274274	chrX	146994128	-	FMR1_promoter_repeat_locus_Hyper_in_male_is_FragileX
cg19741073	chrX	146994166	-	FMR1_promoter_repeat_locus_Hyper_in_male_is_FragileX
cg21378039	chrX	146994369	+	FMR1_promoter_repeat_locus_Hyper_in_male_is_FragileX

Table S2. Accumulative variance explained by principal components calculated from the 109,131 above-background-noise probes among 283 samples included in the studied cohort

	Standard deviation	Proportion of Variance	Cumulative Proportion
PC1	173.387232	0.27548	0.27548
PC2	117.6285937	0.12679	0.40227
PC3	67.2883975	0.04149	0.44376
PC4	61.25697909	0.03438	0.47814
PC5	50.66611742	0.02352	0.50167
PC6	44.55919383	0.01819	0.51986
PC7	36.79718412	0.01241	0.53227
PC8	33.90805961	0.01054	0.5428
PC9	32.17932559	0.00949	0.55229
PC10	30.5450202	0.00855	0.56084
PC11	25.7448014	0.00607	0.56691
PC12	24.59543615	0.00554	0.57246
PC13	22.49945078	0.00464	0.5771
PC14	22.32389153	0.00457	0.58166
PC15	21.8846006	0.00439	0.58605
PC16	21.17879136	0.00411	0.59016
PC17	20.79268859	0.00396	0.59412
PC18	20.48575128	0.00385	0.59797
PC19	20.26531104	0.00376	0.60173
PC20	19.66392731	0.00354	0.60528
PC21	19.58861249	0.00352	0.60879
PC22	19.41353493	0.00345	0.61225
PC23	19.2342608	0.00339	0.61564
PC24	18.92551216	0.00328	0.61892
PC25	18.76216109	0.00323	0.62214
PC26	18.44818157	0.00312	0.62526
PC27	18.28086234	0.00306	0.62832
PC28	18.21369673	0.00304	0.63136
PC29	17.97974043	0.00296	0.63433
PC30	17.88037364	0.00293	0.63726
PC31	17.6153547	0.00284	0.6401
PC32	17.55594476	0.00282	0.64292
PC33	17.36394023	0.00276	0.64569
PC34	17.33118913	0.00275	0.64844
PC35	17.07516042	0.00267	0.65111
PC36	17.02918779	0.00266	0.65377
PC37	16.97491394	0.00264	0.65641
PC38	16.82962118	0.0026	0.659

PC39	16.75559604	0.00257	0.66158
PC40	16.6368798	0.00254	0.66411
PC41	16.62995917	0.00253	0.66665
PC42	16.56610739	0.00251	0.66916
PC43	16.49279604	0.00249	0.67165
PC44	16.41098715	0.00247	0.67412
PC45	16.26134961	0.00242	0.67655
PC46	16.08516746	0.00237	0.67892
PC47	15.98041252	0.00234	0.68126
PC48	15.94195164	0.00233	0.68359
PC49	15.84700496	0.0023	0.68589
PC50	15.81546534	0.00229	0.68818
PC51	15.79281969	0.00229	0.69046
PC52	15.69807125	0.00226	0.69272
PC53	15.59596513	0.00223	0.69495
PC54	15.57726176	0.00222	0.69717
PC55	15.52910924	0.00221	0.69938
PC56	15.45250389	0.00219	0.70157
PC57	15.279597	0.00214	0.70371
PC58	15.23907073	0.00213	0.70584
PC59	15.15135196	0.0021	0.70794
PC60	15.12555423	0.0021	0.71004
PC61	15.04994937	0.00208	0.71211
PC62	15.04533169	0.00207	0.71419
PC63	14.96685741	0.00205	0.71624
PC64	14.95614143	0.00205	0.71829
PC65	14.84215934	0.00202	0.72031
PC66	14.82258127	0.00201	0.72232
PC67	14.8212477	0.00201	0.72434
PC68	14.68207662	0.00198	0.72631
PC69	14.67615926	0.00197	0.72829
PC70	14.64552486	0.00197	0.73025
PC71	14.5437678	0.00194	0.73219
PC72	14.49644242	0.00193	0.73411
PC73	14.4783695	0.00192	0.73604
PC74	14.45330469	0.00191	0.73795
PC75	14.43323895	0.00191	0.73986
PC76	14.39328252	0.0019	0.74176
PC77	14.36665791	0.00189	0.74365
PC78	14.28944543	0.00187	0.74552
PC79	14.24023502	0.00186	0.74738

PC80	14.22945319	0.00186	0.74923
PC81	14.15844645	0.00184	0.75107
PC82	14.12673778	0.00183	0.7529
PC83	14.09923543	0.00182	0.75472
PC84	14.04594935	0.00181	0.75653
PC85	13.98820816	0.00179	0.75832
PC86	13.96925222	0.00179	0.76011
PC87	13.92653134	0.00178	0.76189
PC88	13.9152099	0.00177	0.76366
PC89	13.85471702	0.00176	0.76542
PC90	13.77841524	0.00174	0.76716
PC91	13.75330861	0.00173	0.76889
PC92	13.72769888	0.00173	0.77062
PC93	13.72447645	0.00173	0.77235
PC94	13.70782084	0.00172	0.77407
PC95	13.64427283	0.00171	0.77577
PC96	13.62615001	0.0017	0.77747
PC97	13.606489	0.0017	0.77917
PC98	13.54635877	0.00168	0.78085
PC99	13.53489047	0.00168	0.78253
PC100	13.47276262	0.00166	0.78419
PC101	13.44727121	0.00166	0.78585
PC102	13.39253051	0.00164	0.7875
PC103	13.36562792	0.00164	0.78913
PC104	13.35841525	0.00164	0.79077
PC105	13.28416879	0.00162	0.79238
PC106	13.26297398	0.00161	0.794
PC107	13.25491209	0.00161	0.79561
PC108	13.21894404	0.0016	0.79721
PC109	13.20311698	0.0016	0.7988
PC110	13.17086319	0.00159	0.80039
PC111	13.14227513	0.00158	0.80198
PC112	13.1241569	0.00158	0.80356
PC113	13.1046901	0.00157	0.80513
PC114	13.05078843	0.00156	0.80669
PC115	13.03442541	0.00156	0.80825
PC116	13.00807863	0.00155	0.8098
PC117	12.98732623	0.00155	0.81134
PC118	12.95842021	0.00154	0.81288
PC119	12.93387092	0.00153	0.81441
PC120	12.88978221	0.00152	0.81594

PC121	12.8695209	0.00152	0.81745
PC122	12.85355215	0.00151	0.81897
PC123	12.83177722	0.00151	0.82048
PC124	12.80717233	0.0015	0.82198
PC125	12.76857847	0.00149	0.82347
PC126	12.7403869	0.00149	0.82496
PC127	12.71218768	0.00148	0.82644
PC128	12.67865523	0.00147	0.82792
PC129	12.67703601	0.00147	0.82939
PC130	12.64211413	0.00146	0.83085
PC131	12.63581353	0.00146	0.83232
PC132	12.60724241	0.00146	0.83377
PC133	12.58962252	0.00145	0.83522
PC134	12.57131465	0.00145	0.83667
PC135	12.5324647	0.00144	0.83811
PC136	12.49051114	0.00143	0.83954
PC137	12.44356898	0.00142	0.84096
PC138	12.43813316	0.00142	0.84238
PC139	12.41274081	0.00141	0.84379
PC140	12.3568922	0.0014	0.84519
PC141	12.34743908	0.0014	0.84659
PC142	12.32625536	0.00139	0.84798
PC143	12.29000713	0.00138	0.84936
PC144	12.27147171	0.00138	0.85074
PC145	12.25921788	0.00138	0.85212
PC146	12.24057291	0.00137	0.85349
PC147	12.19226336	0.00136	0.85485
PC148	12.1851113	0.00136	0.85621
PC149	12.17365951	0.00136	0.85757
PC150	12.11814692	0.00135	0.85892
PC151	12.08111747	0.00134	0.86026
PC152	12.0669122	0.00133	0.86159
PC153	12.04916579	0.00133	0.86292
PC154	12.02493019	0.00133	0.86425
PC155	12.01410549	0.00132	0.86557
PC156	11.99229917	0.00132	0.86689
PC157	11.94658945	0.00131	0.86819
PC158	11.93610158	0.00131	0.8695
PC159	11.89604494	0.0013	0.8708
PC160	11.86964043	0.00129	0.87209
PC161	11.85963951	0.00129	0.87338

PC162	11.84982364	0.00129	0.87466
PC163	11.82630165	0.00128	0.87594
PC164	11.81634518	0.00128	0.87722
PC165	11.7739288	0.00127	0.87849
PC166	11.76186944	0.00127	0.87976
PC167	11.74567116	0.00126	0.88103
PC168	11.70054876	0.00125	0.88228
PC169	11.67470614	0.00125	0.88353
PC170	11.66908689	0.00125	0.88478
PC171	11.62559445	0.00124	0.88602
PC172	11.62201559	0.00124	0.88725
PC173	11.61233366	0.00124	0.88849
PC174	11.58338676	0.00123	0.88972
PC175	11.56187721	0.00122	0.89094
PC176	11.54814804	0.00122	0.89217
PC177	11.53477536	0.00122	0.89338
PC178	11.49424092	0.00121	0.8946
PC179	11.46985181	0.00121	0.8958
PC180	11.45547678	0.0012	0.897
PC181	11.43085688	0.0012	0.8982
PC182	11.42254888	0.0012	0.8994
PC183	11.41035263	0.00119	0.90059
PC184	11.39419964	0.00119	0.90178
PC185	11.37088403	0.00118	0.90296
PC186	11.32230549	0.00117	0.90414
PC187	11.3163819	0.00117	0.90531
PC188	11.30193441	0.00117	0.90648
PC189	11.29021446	0.00117	0.90765
PC190	11.27340041	0.00116	0.90881
PC191	11.24141426	0.00116	0.90997
PC192	11.22728951	0.00116	0.91113
PC193	11.20299612	0.00115	0.91228
PC194	11.19924646	0.00115	0.91343
PC195	11.17002212	0.00114	0.91457
PC196	11.14393311	0.00114	0.91571
PC197	11.11838918	0.00113	0.91684
PC198	11.11055044	0.00113	0.91797
PC199	11.10568478	0.00113	0.9191
PC200	11.07513412	0.00112	0.92023
PC201	11.05616217	0.00112	0.92135
PC202	11.05456414	0.00112	0.92247

PC203	11.03887328	0.00112	0.92358
PC204	11.0207352	0.00111	0.9247
PC205	11.00458238	0.00111	0.92581
PC206	10.97817147	0.0011	0.92691
PC207	10.96659784	0.0011	0.92801
PC208	10.9553399	0.0011	0.92911
PC209	10.93523598	0.0011	0.93021
PC210	10.91909097	0.00109	0.9313
PC211	10.88940392	0.00109	0.93239
PC212	10.87875865	0.00108	0.93347
PC213	10.86777066	0.00108	0.93455
PC214	10.84352159	0.00108	0.93563
PC215	10.83553329	0.00108	0.93671
PC216	10.82630905	0.00107	0.93778
PC217	10.80596403	0.00107	0.93885
PC218	10.78368738	0.00107	0.93992
PC219	10.76498238	0.00106	0.94098
PC220	10.74662691	0.00106	0.94204
PC221	10.73792314	0.00106	0.94309
PC222	10.72803811	0.00105	0.94415
PC223	10.70674343	0.00105	0.9452
PC224	10.69712797	0.00105	0.94625
PC225	10.69638064	0.00105	0.9473
PC226	10.65073926	0.00104	0.94833
PC227	10.62728938	0.00103	0.94937
PC228	10.61344605	0.00103	0.9504
PC229	10.58556932	0.00103	0.95143
PC230	10.58266123	0.00103	0.95246
PC231	10.56140543	0.00102	0.95348
PC232	10.54322543	0.00102	0.9545
PC233	10.5333511	0.00102	0.95551
PC234	10.50660282	0.00101	0.95652
PC235	10.49713847	0.00101	0.95753
PC236	10.48366107	0.00101	0.95854
PC237	10.46719819	0.001	0.95954
PC238	10.43340072	0.001	0.96054
PC239	10.41310232	0.00099	0.96154
PC240	10.40108763	0.00099	0.96253
PC241	10.3670578	0.00098	0.96351
PC242	10.35850091	0.00098	0.9645
PC243	10.34481753	0.00098	0.96548

PC244	10.32138922	0.00098	0.96645
PC245	10.30885667	0.00097	0.96743
PC246	10.27930073	0.00097	0.96839
PC247	10.25141918	0.00096	0.96936
PC248	10.2423851	0.00096	0.97032
PC249	10.22600612	0.00096	0.97128
PC250	10.19734779	0.00095	0.97223
PC251	10.16863167	0.00095	0.97318
PC252	10.14742574	0.00094	0.97412
PC253	10.135512	0.00094	0.97506
PC254	10.10521707	0.00094	0.976
PC255	10.07346134	0.00093	0.97693
PC256	10.06907418	0.00093	0.97786
PC257	10.04472857	0.00092	0.97878
PC258	10.03742911	0.00092	0.9797
PC259	9.992419093	0.00091	0.98062
PC260	9.966540557	0.00091	0.98153
PC261	9.947706239	0.00091	0.98244
PC262	9.939802013	0.00091	0.98334
PC263	9.897598589	0.0009	0.98424
PC264	9.872599862	0.00089	0.98513
PC265	9.862600949	0.00089	0.98602
PC266	9.835884979	0.00089	0.98691
PC267	9.804713596	0.00088	0.98779
PC268	9.776460554	0.00088	0.98867
PC269	9.74533231	0.00087	0.98954
PC270	9.726684902	0.00087	0.9904
PC271	9.717883893	0.00087	0.99127
PC272	9.648402372	0.00085	0.99212
PC273	9.640318219	0.00085	0.99297
PC274	9.610641375	0.00085	0.99382
PC275	9.591746717	0.00084	0.99466
PC276	9.568294881	0.00084	0.9955
PC277	9.511352334	0.00083	0.99633
PC278	9.411713705	0.00081	0.99714
PC279	9.294634351	0.00079	0.99793
PC280	9.040597222	0.00075	0.99868
PC281	8.92361337	0.00073	0.99941
PC282	8.001160442	0.00059	1
PC283	1.87E-13	0	1

Table S3. Possibility of each sample in test set being predicted as each of the classes by the classifier trained in this study after adjustment and calibration

	AS2	BWS1	BWS2	FXS_M	Normal	PWS1	PWS2	SRS1
Normal_10Y_1	0.001814	0.002723	0.002112	0.003078	0.98198	0.002093	0.002496	0.003703
Normal_12Y_3	0.007708	0.01242	0.009	0.013273	0.8497	0.028654	0.022337	0.056907
Normal_13Y_1	0.011703	0.013498	0.010907	0.023554	0.874206	0.034776	0.011136	0.020219
Normal_17Y_2	0.009321	0.008916	0.009889	0.006473	0.894991	0.028889	0.009578	0.031944
Normal_18Y_3	0.006575	0.04594	0.007403	0.015174	0.863105	0.030882	0.013123	0.017798
Normal_1Y_1	0.008929	0.01709	0.005902	0.041739	0.869037	0.009265	0.019869	0.028169
Normal_21Y_1	0.008283	0.011151	0.00882	0.007586	0.907552	0.029115	0.012625	0.014869
Normal_21Y_3	0.004056	0.008528	0.004874	0.024468	0.883484	0.013581	0.024582	0.036427
Normal_30Y_1	0.003412	0.005113	0.00345	0.023699	0.951605	0.003711	0.003743	0.005267
Normal_33Y_1	0.008485	0.008201	0.008212	0.006958	0.915371	0.028604	0.009835	0.014334
Normal_33Y_3	0.003485	0.006037	0.003764	0.025481	0.929193	0.012727	0.008729	0.010584
Normal_36Y_2	0.011688	0.021471	0.012035	0.014891	0.818056	0.050127	0.046237	0.025495
Normal_3M_1	0.010177	0.038096	0.015732	0.020134	0.8719	0.018099	0.013199	0.012664
Normal_3Y_2	0.005107	0.004166	0.002738	0.002401	0.973786	0.004101	0.002476	0.005225
Normal_48Y_3	0.00398	0.004131	0.007083	0.005935	0.961166	0.005273	0.005247	0.007184
Normal_54Y_3	0.008924	0.03732	0.009525	0.111593	0.607209	0.047134	0.160373	0.017922
Normal_54Y_6	0.003355	0.004856	0.004848	0.007791	0.944928	0.015423	0.006496	0.012303
Normal_57Y_4	0.001693	0.001967	0.001757	0.00559	0.976824	0.001718	0.0019	0.008552
Normal_57Y_5	0.003226	0.001818	0.001757	0.001601	0.985014	0.0026	0.001765	0.002219
Normal_5Y_3	0.001989	0.011035	0.002058	0.00342	0.967649	0.003172	0.004518	0.006158
Normal_60Y_2	0.003901	0.007372	0.009057	0.019527	0.888238	0.008641	0.016453	0.04681
Normal_63Y_3	0.003172	0.003595	0.003712	0.008135	0.956993	0.00847	0.004879	0.011044
Normal_66Y_4	0.003483	0.003861	0.004008	0.004099	0.969188	0.005434	0.003922	0.006006
Normal_69Y_4	0.005333	0.010155	0.006456	0.021316	0.910818	0.014513	0.019994	0.011414
Normal_6M_1	0.008589	0.007774	0.010144	0.021881	0.886702	0.011052	0.010014	0.043844
Normal_75Y_2	0.001519	0.00152	0.001736	0.001708	0.987033	0.001849	0.001456	0.003179
Normal_7Y_4	0.004068	0.002566	0.002198	0.007532	0.963358	0.003863	0.005288	0.011128
Normal_81Y_2	0.005092	0.023856	0.003172	0.00471	0.929584	0.011746	0.017962	0.003877
Normal_9Y_1	0.002617	0.003419	0.002132	0.003907	0.979802	0.002417	0.002375	0.00333
BWS2_M119	0.024314	0.090502	0.645666	0.023594	0.018697	0.157377	0.013228	0.026623
SRS1_M129	0.069559	0.028296	0.039599	0.011211	0.084323	0.05106	0.091763	0.624189
SRS1_M139	0.007646	0.002008	0.003079	0.002583	0.00643	0.002008	0.002098	0.974147
SRS1_M144	0.087227	0.006597	0.008821	0.004751	0.016151	0.006244	0.008366	0.861842
AS2_M155	0.968581	0.012919	0.00168	0.001095	0.005948	0.001361	0.001717	0.0067
PWS2_M170	0.003024	0.017196	0.007733	0.024027	0.007813	0.056625	0.853984	0.029598
PWS2_M176	0.018784	0.038192	0.009755	0.011991	0.040587	0.323047	0.472412	0.085231
BWS1_M18	0.010362	0.706184	0.022156	0.006924	0.228329	0.009527	0.015119	0.001398
SRS1_M186	0.030056	0.007813	0.007957	0.086317	0.433498	0.014598	0.005876	0.413886

PWS2_M223	0.004136	0.00791	0.008036	0.007207	0.009539	0.167322	0.736636	0.059214
BWS1_M234	0.028131	0.118237	0.812139	0.005572	0.013504	0.003581	0.008095	0.01074
BWS2_M255	0.004415	0.047228	0.921398	0.00182	0.00869	0.005687	0.006931	0.003831
BWS1_M256	0.001737	0.95186	0.024938	0.011958	0.00391	0.001412	0.002204	0.001981
AS2_M258	0.947845	0.013998	0.007993	0.002315	0.009499	0.002025	0.00361	0.012715
BWS2_M261	0.002133	0.01327	0.943621	0.001179	0.029965	0.004631	0.00286	0.00234
SRS1_M264	0.001659	0.001411	0.002053	0.003182	0.006521	0.001417	0.002934	0.980823
FXS_M_M32	0.005869	0.011796	0.002239	0.895592	0.064333	0.004988	0.01158	0.003604
FXS_M_M34	0.022202	0.025043	0.012358	0.590881	0.273903	0.0211	0.038456	0.016057
SRS1_M48	0.009192	0.005368	0.039876	0.01516	0.366124	0.076462	0.012447	0.475371
AS2_M71	0.947727	0.023291	0.002921	0.003168	0.005622	0.001725	0.003049	0.012497
PWS1_M78	0.003154	0.007624	0.01436	0.004478	0.014046	0.858518	0.067589	0.030233
PWS1_M81	0.006349	0.025846	0.029085	0.008938	0.033527	0.774937	0.086457	0.034862
PWS1_M89	0.00208	0.003014	0.007891	0.001866	0.017726	0.947629	0.016264	0.003529
